# Electrophysiological Explorations of the Bilingual Advantage: Evidence from a Stroop Task

**DOI:** 10.1371/journal.pone.0103424

**Published:** 2014-07-28

**Authors:** Emily L. Coderre, Walter J. B. van Heuven

**Affiliations:** School of Psychology, University of Nottingham, Nottingham, United Kingdom; Baycrest Hospital, Canada

## Abstract

Bilinguals have been shown to exhibit a performance advantage on executive control tasks, outperforming their monolingual counterparts. Although a wealth of research has investigated this ‘bilingual advantage’ behaviourally, electrophysiological correlates are lacking. Using EEG with a Stroop task that manipulated the stimulus onset asynchrony (SOA) of word and colour presentation, the current study addressed two facets of the bilingual advantage. The possibility that bilinguals experience superior conflict processing relative to monolinguals (a ‘conflict-specific advantage’) was investigated by comparing behavioural interference effects as well as the amplitude of the N_inc_, a conflict-related ERP component occurring from approximately 300–500 ms after the onset of conflict. In contrast, the hypothesis that bilinguals experience domain-general, conflict-independent enhancements in executive processing (a ‘non-conflict-specific advantage’) was evaluated by comparing the control condition (symbol strings) between groups. There was some significant, but inconsistent, evidence for a conflict-specific bilingual advantage. In contrast, strong evidence emerged for a non-conflict-specific advantage, with bilinguals demonstrating faster RTs and reduced ERP amplitudes on control trials compared to monolinguals. Importantly, when the control stimulus was presented before the colour, ERPs to control trials revealed group differences before the onset of conflict, suggesting differences in the ability to ignore or suppress distracting irrelevant information. This indicates that bilinguals experience superior executive processing even in the absence of conflict and semantic salience, and suggests that the advantage extends to more efficient proactive management of the environment.

## Introduction

‘Executive control’ is an umbrella term comprising a host of functions such as decision making, task switching, and inhibitory control. Inhibitory control in particular is typically studied using paradigms that require overcoming conflict. For example, the Stroop task [1] consists of colour words printed in coloured ink and instructs participants to ignore the word and name its ink colour. In incongruent trials, in which the word and colour do not match (e.g. ‘red’ printed in blue ink, correct response ‘blue’), the conflict between the word and colour stimuli must be resolved before a correct response can be made. Overcoming this conflict creates longer reaction times (RTs) compared to control trials (e.g. ‘xxxx’ printed in blue). In contrast, for congruent trials, in which the word and colour match, the converging information facilitates a response and leads to faster RTs (see [2] for a review). The ‘Stroop effect’ is typically calculated as incongruent RTs minus congruent RTs; ‘interference effects’ as incongruent minus control RTs; and ‘facilitation effects’ as control minus congruent RTs. The magnitudes of Stroop or interference effects are often used as a proxy of executive control abilities, reflecting the success in monitoring for, detecting, and resolving cognitive conflict. Many individual differences can affect executive control abilities (e.g. [3], [4]). One such difference, which is the focus of the current study, is bilingualism.

### The bilingual advantage in executive control: Two hypotheses

A popular phenomenon in the bilingualism literature is that of a ‘bilingual advantage’ in executive control: across a range of executive function tasks, bilinguals routinely outperform their monolingual counterparts (see [5]–[9] for reviews). This is thought to occur because bilinguals activate both of their languages in parallel, even in completely monolingual contexts [10]–[18]. This ‘nonselective’ activation creates cross-linguistic influences, which can cause both interference and facilitation during language processing (see reviews in [19]–[22]). These cross-linguistic influences necessitate a mechanism of language control to avoid cross-linguistic speech or comprehension errors (e.g. [22], [23]; although how and where control is operating might differ for comprehension and production: see [24]). Accordingly, studies of bilingual language processing have demonstrated that bilinguals actively recruit cognitive control during language processing (e.g. [16], [17], [25]). Note that monolinguals also recruit cognitive control during language processing (e.g. [26]), but theoretically bilinguals recruit these functions to a greater extent due to the added cross-linguistic influences from non-selective lexical access [27]. This recruitment of executive control during language processing is thought to enhance executive control abilities, generating a bilingual advantage on executive control tasks.

A bilingual advantage has been documented on a host of cognitive control tasks, including tasks of cognitive flexibility [28]–[32], attentional control [33], [34], and even theory of mind [35], [36] and learning new words [37]. In inhibitory control specifically, bilinguals generally show smaller interference effects (the RT difference between incongruent and control trials) than monolinguals on both linguistic and non-linguistic conflict paradigms like the Stroop and Simon tasks [38]–[45], suggesting superior abilities at managing and resolving domain-general conflict. The smaller conflict effects for bilinguals when comparing incongruent and congruent trials is termed the bilingual ‘interference advantage’.

The bilingual advantage has garnered significant attention in the past few years, as the reliability and robustness of this phenomenon has been called into question in a number of review papers [9], [46], [47]. In a recent review, Hilchey & Klein [9] provided an alternative way of assessing the bilingual advantage. They reported that a more common finding is the presence of a bilingual ‘global reaction time’ advantage such that bilinguals are faster than monolinguals on *all* trial types: incongruent, congruent, and control (e.g. [33], [39], [42], [48]; although see [40]). To distinguish between the bilingual interference advantage and the bilingual global RT advantage, Hilchey & Klein outlined two hypotheses regarding bilingual executive processing: the ‘bilingual inhibitory control advantage’, or BICA, hypothesis; and the ‘bilingual executive processing advantage’, or BEPA, hypothesis.

The *BICA hypothesis* refers specifically to the finding of smaller interference effects on inhibitory control tasks for bilinguals compared to monolinguals. This is thought to occur because bilinguals engage inhibitory control mechanisms to control cross-linguistic interference during language processing (see the Inhibitory Control model: [23]). This therefore predicts that bilinguals will have more efficient inhibitory processes in the presence of conflict, conferring an advantage on incongruent trials and resulting in reduced interference effects compared to monolinguals. Most conflict tasks assess inhibitory control by quantifying and comparing interference effects (incongruent vs. control) and Stroop/Simon effects (incongruent vs. congruent); since the BICA hypothesis predicts superior performance on these measures due to the presence of conflict, this ‘interference advantage’ has become the most common conception of the bilingual advantage. Crucially, as the BICA hypothesis predicts advantages in conflict control, it predicts no difference between groups in the absence of conflict, such as control or congruent trials. It therefore cannot explain why bilinguals more often demonstrate faster RTs on all trial types, i.e. the global RT advantage.

In contrast, the *BEPA hypothesis* states that bilinguals have an advantage in domain-general executive processing which is not restricted just to conflict processing, but is manifest as a more general advantage in cognitive monitoring [33], [42]. Although Hilchey & Klein do not propose a formal model for how this advantage might manifest, evidence from the literature supports this hypothesis and provides a number of mechanisms by which it might work. For example, Costa et al. [33] proposed that bilinguals have a more efficient mechanism of monitoring the environment for conflict and evaluating the need to implement conflict resolution processes. This enhanced domain-general executive processing leads to faster processing in all contexts and on all trial types, thus explaining the global RT advantage. Alternatively, Hernández et al. [49] have suggested that bilinguals have more efficient top-down guidance of attention, allowing them to be less distracted by irrelevant information and more efficient at guiding their attention towards the relevant stimulus. This would explain the global RT advantage even on control trials: although there is no conflict present in control trials, these trials still require executive processes such as attending to one dimension of the stimulus and ignoring the other, thus it may be that bilinguals are better at directing attentional control towards the task-relevant stimulus and away from the task-irrelevant distracting stimulus. Because the global RT advantage generally reduces processing time equally across congruencies, bilinguals and monolinguals may not differ significantly when comparing interference effects, which could explain the elusiveness of the bilingual interference advantage. However, the difference in cognitive abilities can become clear when comparing the global RT (collapsed over congruencies) or control RTs.

The BICA and BEPA hypotheses will be investigated in the current study via investigation of the presence of two specific subtypes of the bilingual advantage. To avoid confusing terminology, we will henceforth use the term ‘conflict-specific advantage’ in reference to the phenomenon that bilinguals might show an advantage in inhibitory control, as proposed by the BICA hypothesis. We will identify a conflict-specific advantage in bilinguals by comparing Stroop (incongruent vs. congruent) and interference (incongruent vs. control) effects. In contrast, we use the term ‘non-conflict-specific advantage’ to refer to the phenomenon that bilinguals show more efficient global cognitive control, as proposed by the BEPA hypothesis. In the current study, we use the control condition as a metric of this advantage to eliminate the influence of conflicting or facilitating information (from incongruent and congruent conditions, respectively) that may contribute to the global RT when collapsing over congruencies. Because the control stimulus in our paradigm (a string of percent signs) contained no linguistic or conflicting information, comparing groups on this neutral condition should provide a better measure of domain-general executive control than collapsing across all congruencies. (In addition, note that the term ‘bilingual advantage’ is used here to more generally refer to advantages on executive control tasks, and comprises both the interference and global advantage. Similarly, the term ‘cognitive control’ is used to describe executive control processes more generally, when we discuss both inhibitory control and general monitoring abilities, or when the specific type of control is not determinable. In contrast, ‘inhibitory control’ refers specifically to the resolution of conflict.).

Note that the BICA and BEPA theories are not mutually exclusive. Although Hilchey & Klein [9] offer no hypotheses regarding the presence of both smaller interference effects and faster overall RTs, it is possible that bilinguals have superior inhibitory control *and* more general cognitive processing abilities, as some researchers have reported (e.g. [44]). We investigate conflict-specific and non-conflict-specific effects independently, but do not rule out the possibility of finding evidence of both. Importantly, however, the main distinction between the BICA and BEPA hypotheses is that the BICA hypothesis predicts group differences *only in the presence of conflict*, whereas the BEPA hypothesis predicts more domain-general advantages in attentional control or monitoring. Therefore situations in which stimulus presentation occurs similarly but the onset of conflict is delayed, the presence of group differences before the onset of conflict would support the BEPA but not the BICA hypothesis. The current study tests this hypothesis by recording electrophysiology (EEG) during a Stroop task with varying SOAs. In the following sections we review the literature on the bilingual advantage using EEG and the manipulation of SOA, respectively.

### Electrophysiological measures of executive control and the bilingual advantage

Behavioural studies are notably limited in their ability to detect subtle differences in cognitive functioning between groups; multiple different underlying cognitive functions could produce similar behavioural results both within and between individuals. For example, fMRI studies have shown that even when monolinguals and bilinguals show no differences in behavioural effects, they show different patterns of brain activity underlying those similar behavioural effects (e.g. [50]). Therefore neuroimaging techniques are crucial for a more fine-grained measure of cognitive differences between groups, to which RT latencies might not be sensitive. By just looking at behavioural results, the bilingual advantage literature may be missing more subtle differences between monolinguals and bilinguals in the underlying cognitive architecture.

The current study used EEG to obtain fine-grained temporal information about the neural activity underlying the bilingual advantage. Although many studies have investigated the bilingual advantage behaviourally, surprisingly few have investigated the neural correlates of this enhanced control. The majority of neuroimaging studies have used fMRI, documenting differences between monolinguals and bilinguals in the recruitment and activation of the executive control network during conflict processing or task-switching [28], [48], [50], [51]. However, as we will describe in more detail below, group differences occurring before the onset of conflict would be critical for distinguishing between the BICA and BEPA hypotheses, yet there is a paucity of literature investigating the bilingual advantage using EEG.

In monolingual studies of conflict processing and executive control, the event-related potential (ERP) of primary interest is a more negative-going wave in incongruent trials as compared to congruent or control trials. In the Stroop task in particular, incongruency effects typically occur from approximately 300–550 ms post-stimulus over centro-parietal scalp [52]–[56]; this component is sometimes referred to as an N400 or N450. We refer to this conflict component in the Stroop task as the N_inc_ (a ‘negativity associated with incongruency’; see [57]) to avoid latency specifications and also to avoid confusion with the N400 component that is typically elicited by language and semantic processing (e.g. [58]). The N_inc_ is believed to reflect conflict processes which are more active in incongruent trials, and has been localized to the anterior cingulate cortex (ACC) in the prefrontal lobe [54]–[56], [59], [60], a structure that is reliably activated by executive control tasks (see [61]–[63] for reviews). Our previous work has demonstrated that the N_inc_ is elicited by conflict even after a response has been made [57], suggesting that, while it may reflect more general conflict processing, it is specifically sensitive to conflict detection.

In the only published ERP study to investigate the bilingual advantage using EEG, Kousaie & Phillips [64] compared bilingual and monolingual performance on Stroop, Simon, and flanker tasks. They evaluated the N2, an earlier conflict component typically reported in flanker and Simon conflict tasks that occurs from approximately 200–300 ms at frontocentral locations [65]–[67]. Kousaie & Phillips reported no bilingual advantage in behavioural interference effects, although differences arose in the ERP data: bilinguals showed a smaller N2 in the Stroop task, but no differences in the Simon or flanker tasks. Although the authors focused on the N2 rather than the N_inc_ component, these results suggest that bilinguals may show reduced amplitudes of conflict-related components compared to monolinguals.

Unpublished data by Heidlmayr, Moutier, Hemforth, and Isel (2012), who tested monolinguals and bilinguals on a Stroop task with EEG, showed similar effects. Although there was no behavioural evidence of a bilingual advantage in inhibitory control, in the EEG data monolinguals demonstrated significant conflict effects at the N_inc_ (referred to as an N400) and a late positive component (LPC), whereas the bilingual L1 showed only a significant N_inc_ effect. Comparisons of the group waveforms suggested that the amplitude of the N_inc_ was reduced for bilinguals relative to monolinguals; however, this was not statistically compared.

The results of these two studies thus suggest that bilinguals may exhibit a reduction in conflict-related ERP amplitudes. This could reflect more efficient conflict processing (e.g. [68]), although a positive relationship has also been documented such that poorer cognitive control is associated with a smaller N_inc_ [69], [70]. However, both of these previous studies focused only on conflict-related ERP components, and did not evaluate domain-general differences in executive control as predicted by the BEPA hypothesis.

The current study contributes to this sparse literature by administering a Stroop task in monolinguals and bilinguals using EEG. Unlike other studies, we compared not only conflict effects between groups but also behavioural and electrophysiological responses to the control conditions (symbol strings), thereby specifically evaluating the BICA and BEPA hypotheses. The BICA hypothesis theorizes a conflict-specific bilingual advantage and consequently would predict amplitude differences in the N_inc_ component (as this component is identified by the behaviour of incongruent trial waveforms). Specifically, based on the findings of previous work using EEG to investigate the bilingual advantage, bilinguals are expected to show smaller N_inc_ effects (i.e. a smaller amplitude difference between incongruent and congruent/control waveforms) than monolinguals, demonstrating more efficient processing. In contrast, the BEPA hypothesis predicts a non-conflict-specific bilingual advantage in general executive processing, which we examine by comparing the control trials between groups. In the current paradigm, the control trials contained no linguistic or conflicting semantic information; therefore any differences between the groups on these trials would indicate conflict-independent effects of bilingualism on executive processing. The BEPA hypothesis predicts faster behavioural RTs on control trials for bilinguals; however, if reduced ERP amplitudes are indicative of more efficient processing (e.g. [68]), reduced amplitudes for control waveforms are also predicted in the EEG data. In contrast, the BICA would predict no differences between monolinguals and bilinguals on control trials, either behaviourally or electrophysiologically, as no conflict is present. In particular, the comparison of control trials between monolinguals and bilinguals with EEG will provide valuable insight into the nature of the bilingual global RT advantage.

### SOA manipulation in the Stroop task

The current paradigm also manipulates the stimulus onset asynchrony, or SOA, between the word and the colour stimuli. SOA manipulation provides further tests of the BICA and BEPA hypotheses by potentially eliciting group differences before the onset of conflict (−400 SOA), and by maximizing the amount of inhibitory control required (0 SOA), respectively.

**Figure 1:**
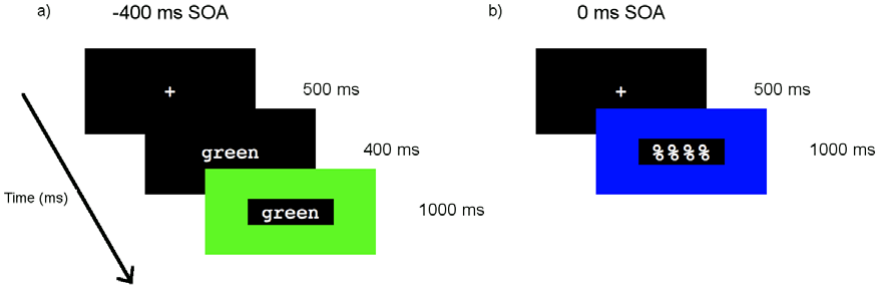
Example trial sequence. Example of the trial sequence for a) a **−**400****ms SOA congruent trial; and b) a 0****ms SOA control trial.

First introduced in the 1970’s and 1980’s [71]–[74], SOA manipulation in the Stroop task varies the onset of word and colour stimuli such that the irrelevant stimulus is either pre- or post-exposed relative to the target stimulus. A ‘negative SOA’ presents the irrelevant stimulus (e.g. the word, in a colour-naming Stroop task) before the relevant target stimulus (the colour) at a specific interval. For example, a negative 400 ms SOA (‘−400 ms SOA’) pre-exposes the word for 400 ms before the colour appears. A ‘positive SOA’ presents the irrelevant stimulus after the relevant: for example, a +400 ms SOA presents the word 400 ms after colour presentation. A ‘0 ms SOA’ presents the word and colour simultaneously, as in a traditional Stroop task (see Figure 1 for an illustration). In a series of seminal experiments, Glaser & Glaser [73] used nine SOAs (−400 ms to +400 ms in 100 ms intervals) to investigate the precise timing of interference. Maximal interference effects occurred at and around the 0 ms SOA; with increasing negative SOAs, interference effects decreased but were not abolished, indicating that word reading can interfere with colour naming even at long pre-exposures. Facilitation effects were larger at negative SOAs than at positive SOAs, indicating beneficial effects of word pre-exposure on congruent trials. In positive SOAs, interference was diminished but still significant at +200 ms, but all effects were gone by later SOAs, indicating that the irrelevant word appears too late to influence colour naming. Studies using the Stroop SOA paradigm with EEG have demonstrated that the N_inc_ occurs earlier in negative SOAs [52], [57], [75]. However, across this previous research, both the −200 ms and −400 ms SOAs show a similar forward shift of approximately 200 ms. This demonstrates that conflict detection processes depend on the speed of lexico-semantic access [57]: Stroop conflict originates at the semantic activation of the irrelevant word, meaning that conflict cannot be detected until both stimuli are presented and the word is fully activated. As full semantic access may occur as early as 200 ms [76], [77], any further pre-exposure beyond approximately 200 ms (i.e. in the −400 ms SOA) does not additionally speed up conflict detection processes.

Importantly, the fact that different amounts of interference are typically elicited by each SOA suggests differences in how the brain exerts inhibitory control in each condition. SOA manipulation thus introduces a manipulation of task difficulty, which may modulate the presence or magnitude of the bilingual interference advantage (e.g. [33], [42]). Although a bilingual advantage has been reported across the lifespan [39], [40], [78]–[81], it seems to be more difficult to elicit in young adults who are at the peak of their cognitive abilities. In young adults, a bilingual advantage is often only found in situations of high cognitive demand (e.g. [33]). As a result, many studies vary the task difficulty by manipulating the proportion of incongruent and congruent trials [33] or the time given to resolve conflict before responding [42]. To maximize the amount of conflict and therefore increase the chances of finding a group difference in inhibitory control that would support the BICA hypothesis, the current study includes a 0 ms SOA. Based on previous Stroop literature using SOA manipulation (e.g. [57], [73]), we predict that the 0 ms SOA will be the most cognitively demanding and will subsequently elicit the largest interference effects in both groups. Based on previous bilingual and ERP literature, we predict that the bilingual interference advantage will be manifest as smaller interference effects (especially in the most cognitively demanding tasks, i.e. the 0 ms SOA) and reduced N_inc_ amplitudes for bilinguals.

To test the BEPA hypothesis, the current study also includes a −400 ms SOA. In the −400 ms SOA, the distracting word or control stimulus is presented 400 ms before the target colour. If, as proposed by the BEPA hypothesis and by others [33], [49], the bilingual advantage stems from more efficient cognitive monitoring and/or guidance of attention, even in the absence of conflict, we might expect group differences after the onset of the distractor but *before* the onset of the target stimulus. In contrast, the BICA hypothesis, which proposes that bilinguals have superior inhibitory control *in the presence of conflict* would predict no differences before the onset of the second stimulus in the −400 ms SOA because conflict cannot occur until both stimuli are presented. We will evaluate this possibility by looking for group differences in the −400 ms SOA before the presentation of the colour stimulus. The use of SOA manipulation in combination with EEG thus provides a novel way of investigating the bilingual advantage.

### The current study

In sum, the current study seeks to evaluate the effects of bilingualism on electrophysiological measures of a Stroop task with SOA manipulation. Specifically, two hypotheses of bilingual executive processing are evaluated. The BICA hypothesis proposes that bilinguals have superior abilities in detecting and resolving conflict and therefore predicts smaller interference effects and reduced N_inc_ amplitudes for bilinguals, particularly in the most cognitively-demanding 0 ms SOA. The BEPA hypothesis predicts that bilinguals experience enhanced executive processing independent of the presence of conflict, which should be reflected in faster RTs and differences in ERP amplitude for control trials. Furthermore, group differences arising before the onset of the target stimulus in the −400 ms SOA would suggest that this domain-general enhancement in executive control stems more specifically from enhanced abilities of monitoring and/or attentional control, in support of the BEPA hypothesis.

To test these hypotheses, we administered a Stroop task with two SOAs to Chinese-English bilinguals and English monolinguals during concurrent EEG recording. Bilinguals performed the Stroop task in both their L1 (Chinese) and their L2 (English) during separate recording sessions. We tested the bilinguals in both languages to rule out the possibility that language-specific characteristics might contribute to any differences between the groups. Importantly, if the experience of bilingualism enhances inhibitory control and executive functions globally, then we hypothesize that a bilingual advantage should occur in both the L1 and L2 for bilinguals compared to monolinguals [43].

It is possible that the L1 and L2 will elicit differences in Stroop interference effects and control RTs due to the relative proficiencies of the languages in bilinguals. For example, previous literature has demonstrated smaller Stroop interference effects in the L2 than in the L1 in bilinguals [82]–[86]. This has been attributed to a ‘reduced automaticity’ of the L2 ([22], [87]; see also the Discussion); in other words, bilinguals experience smaller interference effects in the L2 than in the L1 not because of enhanced cognitive control, but because of a smaller influence of the distracting word. Similarly, although we test the BEPA hypothesis in the current study by comparing control trials, which contain no linguistic information, it is possible that the language context (L1 or L2) may influence domain-general cognitive processing abilities. Crucially, however, for both conflict-specific and non-conflict-specific effects, there should be significant differences for both the bilingual L1 and L2 compared to monolinguals, demonstrating a more global cognitive advantage across languages. Therefore in the current study we hypothesized that a conflict-specific bilingual advantage would be manifest as smaller Stroop interference effects (both behaviourally and electrophysiologically) for both the bilingual L1 and L2 compared to monolinguals, with potential differences between L1 and L2 interference effects. We hypothesized that a non-conflict-specific bilingual advantage would be manifest as smaller control RTs and differences in ERP amplitudes on control trials for both the bilingual L1 and L2 compared to monolinguals.

## Methods

### Participants

#### Bilinguals

The bilingual participants were 25 Chinese-English bilinguals from the University of Nottingham (19 female; mean age = 23.4 years, *SD* = 4.0). All participants reported no colour-blindness and were right-handed. All attended the University of Nottingham as either undergraduate or postgraduate students; they had an average of approximately 17 years of education (M = 16.6, *SD* = 1.7 years). All participants were native Mandarin speakers who had acquired English at approximately 10 years of age (*SD* = 3.4 years) but were dominant in Mandarin. All lived in England at the time of testing and were therefore immersed in their L2 and encountered it on a daily basis. The foreign-language minimum requirements for the University of Nottingham are an IELTS score of 6.0 or a TOEFL score of 79; therefore all participants were proficient in English.

Participants also completed a language background questionnaire prior to testing. Their overall self-reported Chinese proficiency, averaged across reading, writing, speaking and listening, was 9.6 on a 10-point scale (*SD* = 0.9). Their overall self-reported English proficiency, averaged across reading, writing, speaking and listening, was 7.0 on a 10-point scale (*SD* = 1.3). They rated their daily use of L1 as 3.8 (*SD* = 1.0), on a scale from 1 (rarely) to 5 (always), and daily use of L2 as 2.7 (*SD* = 0.9), indicating that they used Chinese more often than English. Some participants (N = 16) reported knowing other languages in addition to Chinese and English. A handful of participants (N = 6) were from Malaysia and also reported speaking Malay. Critically, however, all participants reported that Chinese was the language they felt most comfortable speaking, and the general patterns of results were similar when analysing just participants from mainland China (N = 19).

#### Monolinguals

The monolingual participants were 28 monolingual native English speakers from the University of Nottingham (16 female; mean age = 22.2 years, *SD* = 5.4). All participants reported no colour-blindness and were right-handed. All participants attended the University of Nottingham as students or worked as administrators; they had an average of approximately 15 years of education (M = 15.4, *SD* = 1.7 years). A language background questionnaire was administered before testing to gather more information about native and foreign language skills. The majority of participants (N = 24) reported studying other languages, but none considered themselves proficient in anything but English (average overall proficiency in other languages = 4.12, *SD* = 1.6, on a scale from 1 (not proficient at all) to 10 (fluent)).

The groups did not significantly differ on age (*p* = 0.42), although bilinguals did have slightly more education than monolinguals (*p* = 0.004) due to the fact that the monolingual group consisted of more undergraduate students.

The data from the monolingual participants has been previously reported in [57], which focused on the effects of SOA manipulation on conflict-related ERP components in monolinguals. The data has been re-analysed for the purpose of this comparison with bilinguals.

#### Design and Materials

In the English task, word stimuli consisted of the words ‘red’, ‘green’, and ‘blue’ in lowercase letters printed in white ink on a black background. In the Chinese task, word stimuli were the Chinese characters for ‘red’, ‘green’ and ‘blue’: 红, 绿, and 蓝, printed in white ink on a black background. In both tasks, a non-colour, non-word stimulus that matched the visual input of the words (‘%%%%’in English; ‘%’ in Chinese) was included as a control stimulus, also printed in white ink on a black background. Colour stimuli for both tasks were red, green and blue rectangles surrounding the word stimuli. Participants responded to the colour of the rectangle by pressing a button on the keyboard (right index finger for red, right middle finger for green, right ring finger for blue).

#### Procedure

All procedures were approved by the Ethics Committee of the University of Nottingham School of Psychology. Participants were fully informed of the procedures and the goal of the study, and gave their written informed consent prior to testing.

Bilingual participants performed two sessions, one for each language (L1 Chinese and L2 English), on consecutive days. The order of language administration was counterbalanced across participants. Monolingual participants performed only one session (in English). All testing sessions were approximately 1.5 hours including EEG net application and set-up. Participants were given a brief practice session with only colour stimuli before each session to practise the colour-response mappings, followed by the experimental session which was approximately 50 minutes long.

Stimuli were presented using E-Prime. The experimental session included twelve blocks of approximately 4 minutes each. Two SOAs (−400 ms, 0 ms) were used: SOA was blocked and counterbalanced across participants. (A +400 ms SOA was also included in this experiment (see Coderre, 2012 (unpublished doctoral dissertation)); however, this data is not reported here because only the −400 and 0 SOAs are relevant for investigating the BEPA and BICA hypotheses.) Each SOA included 216 randomly presented trials (72 congruent, 72 control, 72 incongruent). In each trial, a fixation cross first appeared for 500 ms followed by a blank screen for 300 ms. In the −400 ms SOA, the word appeared on the screen alone for 400 ms before being surrounded by the coloured rectangle (Figure 1a). In the 0 ms SOA, both stimuli appeared simultaneously (Figure 1b). Once both word and colour stimuli had appeared, both remained on the screen for 1000 ms, followed by a blank screen presented at an inter-stimulus interval (ISI) varying from 1500–2000 ms before the start of the next trial.

### Data Acquisition

High-density ERPs were recorded at 250 Hz using an EGI Geodesics 128-channel sensor net and NetStation version 4.3. Impedences were kept under 50 kΩ, where possible. Data was preprocessed using EEGlab version 6.0 and Matlab version 7.9. The data was first filtered using a 0.5–40 Hz bandpass filter, and transformed using an average reference transform to the Cz electrode. Correction for eye movement artifacts was performed using a combination of principal component analysis (PCA) and independent component analysis (ICA). First, a PCA was run to identify the number of components required to explain 99% of the data. ICA was then performed using the number of components specified by the PCA. Following ICA decomposition, eye movements, blinks and other noise components were visually identified and manually removed from the data.

The resulting cleaned continuous data was segmented into epochs time-locked to the onset of the colour stimulus. Segments in the −400 ms SOA extended from 500 ms before to 1000 ms after the colour stimulus, in order to include the cognitive response to the word (presented at −400 ms). Segments in the 0 ms SOA extended from 100 ms before to 1000 ms after stimuli presentation. Additional bad epochs were identified and rejected using a joint probability computation. Segments in which the behavioural response was an error or outlier (RTs below 250 ms or above 2000 ms) were also rejected. The resulting segments were baseline corrected using data from the first 100 ms of the segment. For the bilingual L1 there was an average of 94% of trials retained, with an average of 67 trials per trial type in the final analysis. In the L2 there was an average of 94% of trials retained, with an average of 68 trials per trial type. For monolinguals there was an average of 91% of trials left, with an average of 66 trials per trial type in the final analysis.

### Statistical Analysis of EEG Data

Based on previous literature, the N_inc_ occurs from approximately 300–600 ms, so analyses were restricted to this pre-specified time window for the 0 ms SOA. Based on previous literature [52], in the −400 ms SOA the analysis window was defined as the traditional window plus a 200-ms negative shift, making an N_inc_ window from 100–600 ms after presentation of the colour stimulus.

As N_inc_ effects are generally reported at centro-parietal midline electrodes (e.g. [52], [54], [60]), analyses were performed at Cz and Pz. (Analyses were also run using clusters of electrodes around Cz and Pz, and the results were similar.) N_inc_ windows were defined by visual inspection of the data at these electrodes and based on significance using running *t*-tests within the analysis windows defined above. Running *t*-tests were performed by collapsing the raw data into 24 ms bins with 12 ms overlap. Within each bin, the average amplitude was compared between congruencies using paired-sample *t*-tests. All graphs present significant windows (*p*<0.05) of running *t*-tests only within the range of analysis windows (100–800 ms after the second stimulus). In the running *t*-tests, a significant Stroop effect for the N_inc_ was identified as a more negative waveform for incongruent than congruent conditions; interference effects as a more negative waveform for incongruent than control conditions; and facilitation effects as a more negative waveform for control than congruent conditions. As the N_inc_ is a conflict-related component, its presence was identified by a significant difference in the incongruent compared to either the control or congruent conditions; if no significant Stroop or interference effect occurred within a window this was said to be a non-significant effect.

Difference waves (incongruent minus congruent, to eliminate the influence of language processing) were compared between groups using running *t*-tests to identify amplitude differences. Topographic plots of the difference waves are also presented in Figures 4–8 for the relevant N_inc_ windows in each group, to show the extent of component polarity. Electrodes are marked which showed a significant difference (*p*<0.05) in the amplitude of incongruent and congruent trial waveforms averaged over the component window. To identify shifts in the N_inc_ latencies between SOAs, latency analyses were performed on the difference waves within the relevant windows by identifying where the peak minimum amplitude occurred at Cz and Pz, and averaging the peak latency over these electrodes. The averaged peak latencies were then compared using *t*-tests to identify significant effects.

## Results

Incorrect responses (4.1% for monolinguals, 3.1% for L1, 2.7% for L2) and outliers (less than 250 ms or greater than 2000 ms: 0.10% for monolinguals, 0.06% for bilingual L1, 0.11% for bilingual L2) were removed from both the behavioural and ERP data before statistical analysis. The mean number of errors per trial type ranged between 0.5%–1.2% for monolinguals, between 0.3%–0.9% for the bilingual L1, and between 0.1%–0.6% for the bilingual L2. For all analyses, effect sizes are reported in Cohen’s d (for *t*-tests) and eta-squared (η^2^, for ANOVAs) and were calculated accordingly for within-subject or between-subject comparisons.

### RT analyses

**Figure 2:**
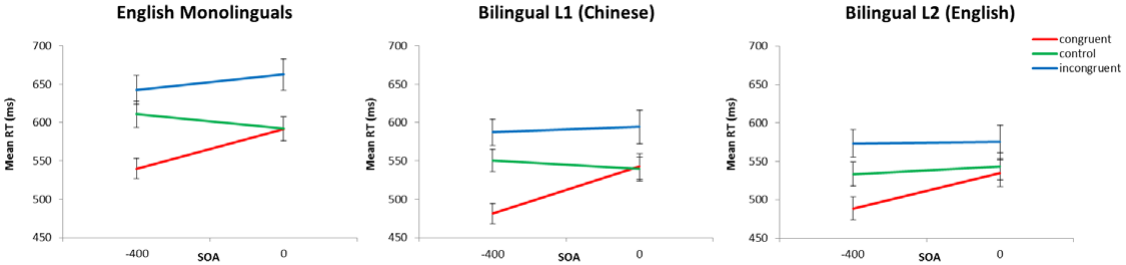
Mean RTs (ms) for each group and language.

The mean RTs for each group are presented in Figure 2. For each group a 2 (SOA)×3 (congruency) repeated-measures ANOVA was run, and follow-up paired-sample *t*-tests with Bonferroni corrections identified significant Stroop, inhibition and facilitation effects.

In monolinguals, there were significant main effects of SOA (*F*(1,27) = 4.77, *p*<0.05, η^2^ = 0.01) and congruency (*F*(2,54) = 69.15, *p*<0.0001, η^2^ = 0.15), and an interaction of SOA and congruency (*F*(2,54) = 27.79, *p*<0.0001, η^2^ = 0.03). Significant Stroop and interference effects occurred at the −400 ms and 0 ms SOAs (see Table 1 for full results). Significant facilitation occurred at the −400 ms SOA only.

**Table 1:** RT analyses: Statistical comparisons of Stroop, interference, and facilitation effects, after Bonferroni-corrections, for each group and language.

Group	Language	SOA	Stroop				Interference				Facilitation			
			*t*-value	*df*	*p*-value	Cohen’s d	*t*-value	*df*	*p*-value	Cohen’s d	*t*-value	*df*	*p*-value	Cohen’s d
Monolinguals	English	−400 ms	9.80	27	<0.0001	2.12	4.06	27	<0.01	0.78	9.30	27	<0.0001	2.06
		0 ms	8.00	27	<0.0001	1.76	6.32	27	<0.0001	1.31	0.06	27	1.00	0.01
Chinese-	Chinese	−400 ms	8.51	24	<0.0001	1.76	4.87	24	<0.001	1.03	8.74	24	<0.0001	1.77
English	(L1)	0 ms	5.56	24	<0.0001	1.34	5.02	24	<0.001	1.22	0.61	24	1.00	0.13
bilinguals	English	−400 ms	9.34	24	<0.0001	2.00	5.70	24	<0.001	1.26	8.00	24	<0.0001	1.56
	(L2)	0 ms	4.02	24	<0.01	0.90	3.30	24	<0.05	0.72	1.85	24	0.46	0.37

In the bilingual L1 there were main effects of SOA (*F*(1,24) = 5.33, *p*<0.05, η^2^ = 0.01) and congruency (*F*(2,48) = 64.19, *p*<0.0001, η^2^ = 0.15) and an interaction (*F*(2,48) = 19.66, *p*<0.0001, η^2^ = 0.04). Significant Stroop and interference effects occurred at −400 ms and 0 ms SOAs (Table 1), and significant facilitation at −400 ms.

The bilingual L2 showed a trend of a main effect of SOA (*F*(1,24) = 3.48, *p* = 0.07, η^2^ = 0.01), a main effect of congruency (*F*(2,48) = 49.45, *p*<0.0001, η^2^ = 0.09) and an interaction of SOA and congruency (*F*(2,48) = 11.68, *p*<0.0001, η^2^ = 0.01). Significant Stroop and interference effects occurred at −400 ms, with a trend of a significant Stroop effect at 0 ms SOA (Table 1), and significant facilitation occurred at −400 ms.

Therefore the patterns of Stroop interference and facilitation effects in a Stroop SOA task for monolinguals replicated previous findings [43], [73]. Similarly, the bilinguals, in both L1 and L2, showed comparable conflict and facilitation patterns as previously reported [43], [57].

#### Comparisons of conflict and facilitation effects between groups

**Figure 3:**
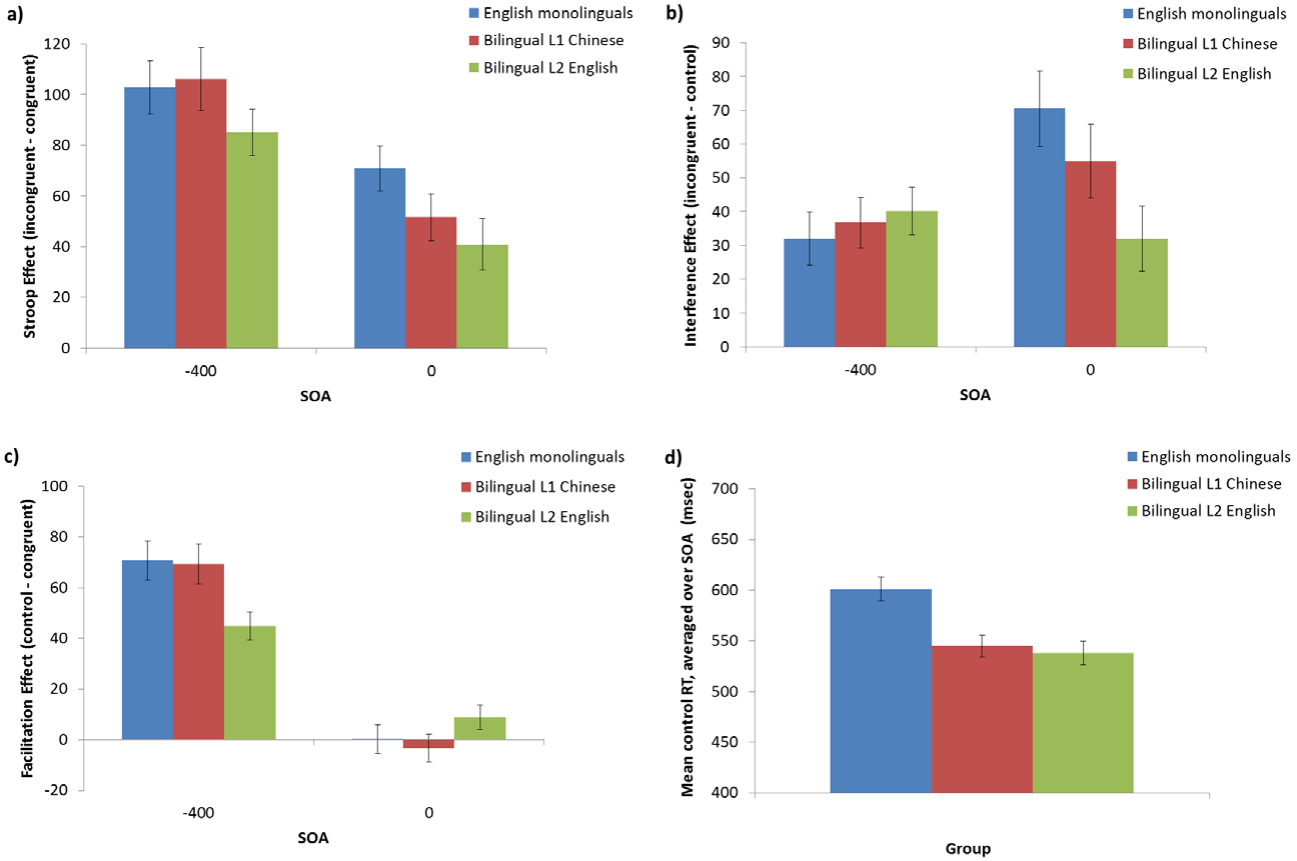
Behavioural data. Behavioural a) Stroop; b) interference; and c) facilitation effects, and d) the average RTs for control trials averaged over SOAs, for the monolinguals, bilingual L1 and bilingual L2.

As stated in the Introduction, we tested the BICA hypothesis by comparing conflict effects between groups. It was not possible to conduct an overall ANOVA with group as a factor with this particular dataset because the bilingual languages were within-subjects. Therefore to compare the groups on the magnitudes of Stroop, interference and facilitation effects, three separate 2 (SOA)×2 (group) ANOVAs were performed for each effect to compare: 1) English monolinguals vs. bilingual L1 (Chinese); 2) English monolinguals vs. bilingual L2 (English); and 3) bilingual L1 (Chinese) vs. bilingual L2 (English). The magnitudes of Stroop, interference and facilitation effects are presented in Figure 3, and full statistical results are presented in Table 2. As we are interested in group effects, only significant main effects of group or interactions with group were followed up with appropriate *t*-tests.

**Table 2:** RT analyses: Results of the 2 (SOA)×2 (group) ANOVAs for each effect and group comparison.

Group comparison	Effect of interest	Main effects/interactions		
		SOA	Group	SOA*group
monolinguals vs.	Stroop	*F*(1,102) = 17.05	*F*<1	*F*(1,102) = 1.19,
bilingual L1		*p*<0.0001		*p* = 0.28
		η^2^ = 0.14		
	Interference	*F*(1,102) = 9.16	*F*<1	*F*(1,102) = 1.11,
		*p*<0.01		*p* = 0.29
		η^2^ = 0.08		
	Facilitation	*F*(1,102) = 111.67	*F*<1	*F*<1
		*p*<0.0001		
		η^2^ = 0.52		
monolinguals vs.	Stroop	*F*(1,102) = 15.16	*F*(1,102) = 6.06	*F*<1
bilingual L2		*p*<0.001	*p*<0.05	
		η^2^ = 0.12	η^2^ = 0.05	
	Interference	*F*(1,102) = 3.32	*F*(1,102) = 2.75	*F*(1,102) = 6.47
		*p* = 0.08	*p* = 0.10	*p*<0.05
		η^2^ = 0.03	η^2^ = 0.02	η^2^ = 0.06
	Facilitation	*F*(1,102) = 78.86	*F*(1,102) = 2.02	*F*(1,102) = 7.96
		*p*<0.0001	*p* = 0.16	*p*<0.01
		η^2^ = 0.41		η^2^ = 0.04
bilingual L1	Stroop	*F*(1,24) = 21.76	*F*(1,24) = 6.17	*F*<1
vs. L2		*p*<0.01	*p*<0.05	
		η^2^ = 0.31	η^2^ = 0.04	
	Interference	*F*<1	*F*(1,24) = 1.60	*F*(1,24) = 4.22
			*p* = 0.22	*p* = 0.05
				η^2^ = 0.04
	Facilitation	*F*(1,24) = 65.76	*F*(1,24) = 1.13	*F*(1,24) = 9.94
		*p*<0.0001	*p* = 0.30	*p*<0.01
		η^2^ = 0.60		η^2^ = 0.17

For monolinguals vs. bilinguals L1, there were no main effects of group and no significant interactions of SOA and group for any effect.

For the monolinguals vs. the bilingual L2, there was a significant interaction of SOA and group for interference effects. Investigating this interaction, monolinguals showed significantly larger interference effects at the 0 ms SOA (monolinguals = 70 ms, *SE* = 11 ms; bilingual L2 = 32 ms, *SE* = 10 ms; *t*(50.6) = 2.61, *p*<0.01, d = 0.72) but there was no group difference at the −400 ms SOA (*t*(50.86)<1, *p* = 0.45). The monolinguals vs. bilingual L2 also showed a significant interaction of group and SOA in facilitation effects. Following up this interaction, monolinguals showed significantly larger facilitation effects than the bilingual L2 at the −400 ms SOA (monolinguals = 71 ms, *SE* = 8 ms; bilingual L2 = 45 ms, *SE* = 6 ms; *t*(48.27) = 2.74, *p*<0.01, d = 0.75) but there was no difference at the 0 ms SOA (*t*(50.5) = 1.15, *p* = 0.25).

For the bilingual L1 vs. L2, there was an interaction of SOA and group in interference and facilitation effects. Interference effects in the 0 ms SOA were marginally significantly larger for the L1 than the L2 (L1 = 55 ms, *SE* = 11 ms; L2 = 32 ms, *SE* = 10 ms; *t*(24) = 1.90, *p* = 0.07, d = 0.38) but there were no significant differences in the −400 ms SOA (*t*(24)<1, *p* = 0.65). Facilitation effects in the −400 ms SOA were significantly larger in the L1 (69 ms, *SE* = 8 ms) than the L2 (45 ms, *SE* = 6 ms; *t*(24) = 3.01, *p*<0.01, d = 0.62), but there was no difference in the 0 ms SOA (*t*(24) = 1.48, *p* = 0.15).

As we were also expecting differences in control RTs between groups, it is possible that these global RT differences might affect the magnitude of conflict and facilitation effects. To account for this possibility, we also ran these analyses using normalized effect scores. Normalized scores were calculated by dividing the original effect size by the RT of the control condition: e.g. normalized interference = (incongruent – control)/control [88], [89]. For all effects, these analyses showed the same patterns of significance as the analyses presented here.

Therefore there were smaller conflict effects for the bilingual L2 compared to monolinguals but not for the bilingual L1 compared to monolinguals. Within bilinguals, interference effects at the 0 ms SOA were slightly larger for the L1 than the L2.

#### Comparisons of non-conflict-specific effects between groups

To investigate the presence of a bilingual non-conflict-specific bilingual advantage, we compared the control RTs between groups. As described in the Introduction, because these trials contained no conflict, they therefore should not show any differences between groups according to the BICA hypothesis. Three 2 (SOA)×2 (group) ANOVAs were run comparing: 1) monolinguals vs. bilingual L1; 2) monolinguals vs. bilingual L2; 3) bilingual L1 vs. L2. Only main effects of group or interactions with group were followed up with appropriate *t*-tests.

For the monolinguals vs. bilingual L1, the 2 (SOA)×2 (group) ANOVA showed a main effect of group (*F*(1,102) = 12.28, *p*<0.001, η^2^ = 0.11) such that monolinguals showed significantly longer control RTs (601 ms, *SE* = 12 ms), collapsed over SOA, than the bilingual L1 (545 ms, *SE* = 11 ms; *t*(103.8) = 3.55, *p*<0.001, d = 0.98; see Figure 3d), but no main effect of SOA (*F*<1) and no interaction (*F*<1). For the monolinguals vs. bilingual L2, there was a main effect of group (*F*(1,102) = 14.06, *p*<0.001, η^2^ = 0.12) such that monolinguals again showed longer control RTs, collapsed over SOA (601 ms, *SE* = 12 ms), than the bilingual L2 (538 ms, *SE* = 12 ms; *t*(103.7) = 3.78, *p*<0.001, d = 1.04; see Figure 3d), but no effect of SOA (*F*<1) or interaction (*F*<1). For the bilingual L1 vs. L2, there was no main effect of language (*F*<1) or SOA (*F*<1) and no interaction (*F*(1,24) = 2.76, *p* = 0.11). Therefore both the bilingual L1 and L2 showed faster control RTs compared to monolinguals, but there was no difference between the L1 and L2. Note that this pattern of results was very similar when comparing the global RTs (i.e. collapsed over all congruencies) between groups, suggesting that this is truly a global advantage.

### Error analyses

To compare error rates within groups, a 2 (SOA)×3 (congruency) repeated-measures ANOVA was run for each group. The monolinguals showed a main effect of SOA (*F*(1,27) = 8.33, *p*<0.01), a main effect of congruency (*F*(2,54) = 6.46, *p*<0.01) and an interaction (*F*(2,54) = 6.17, *p*<0.01). Breaking up this interaction by SOA revealed that there was no main effect of congruency at the −400 ms SOA (*F*<1) but there was a main effect of congruency at the 0 ms SOA (*F*(2,54) = 9.24, *p*<0.001). Follow-up paired *t*-tests showed that there were significantly more errors in the incongruent condition (M = 5.2, *SE* = 0.9) compared to both the control (M = 2.9, *SE* = 0.5; *t*(27) = 2.48, *p*<0.05) and congruent conditions (M = 2.3, *SE* = 0.4; *t*(27) = 4.02, *p*<0.001), but no differences between the congruent and control conditions (*t*(27) = 1.65, *p* = 0.11). Therefore the monolinguals made the most errors in the incongruent condition of the 0 ms SOA, which confirms our predictions that this SOA would be the most cognitively demanding.

In the bilingual L1, the 2×3 ANOVA showed no main effect of SOA (*F*(1,24) = 1.28, *p* = 0.27) a main effect of congruency (*F*(2,48) = 9.84, *p*<0.0001), and no interaction (*F*<1). Follow-up *t*-tests demonstrated that there were more errors, when collapsed over SOA, in the incongruent condition (M = 3.7, *SE* = 0.6) than in the control (M = 1.3, *SE* = 0.2; *t*(49) = 4.71, *p*<0.0001) and the congruent conditions (M = 1.7, *SE* = 0.3; *t*(49) = 3.80, *p*<0.001), but no differences between the congruent and control conditions (*t*(49) = 1.29, *p* = 0.20). Therefore in the bilingual L1, the incongruent condition elicited more errors overall.

In the bilingual L2, there was a main effect of congruency (*F*(2,48) = 23.97, *p*<0.0001). There was no effect of SOA (*F*<1) but a marginal interaction between congruency and SOA (*F*(2,48) = 2.90, *p* = 0.06). Following up this interaction, there was a significant effect of congruency in the −400 ms SOA (*F*(2,48) = 32.68, *p*<0.0001) such that the incongruent condition had significantly more errors (M = 3.7, *SE* = 0.4) than both the control (M = 0.9, *SE* = 0.2; *t*(24) = 6.95, *p*<0.0001) and congruent conditions (M = 1.0, *SE* = 0.2; *t*(24) = 5.96, *p*<0.0001). There were no differences between control and congruent conditions (*t*(24) = 0.26, *p* = 0.80). In the 0 ms SOA there was also a main effect of congruency (*F*(2,48) = 7.72, *p*<0.01) such that the incongruent condition again elicited more errors (M = 3.1, *SE* = 0.7) than the control (M = 1.7, *SE* = 0.3; *t*(24) = 2.31, *p*<0.05) and the congruent conditions (M = 1.1, *SE* = 0.3; *t*(24) = 3.52, *p*<0.01), with no difference between congruent and control (*t*(24) = 1.66, *p* = 0.11). Therefore in the bilingual L2 the incongruent condition in the −400 ms SOA elicited the most errors.

To compare error rates between groups, three 2 (SOA)×3 (congruency)×2 (language) ANOVAs were run to compare: 1) the monolinguals vs. bilingual L1; 2) monolinguals vs. bilingual L2; and 3) bilingual L1 vs. L2. The full results can be found in Table 3. There were significant main effects of group when comparing the monolinguals to the bilinguals in both L1 and L2, but no differences between bilingual L1 and L2. Follow-up *t*-tests indicate that monolinguals had more errors overall, when collapsing over SOA and congruency (M = 3.0, *SE* = 0.2) than the L1 (M = 2.2, *SE* = 0.2; *t*(314.0) = 2.16, *p*<0.05, d = 0.59) and the L2 (M = 1.9, *SE* = 0.2; *t*(302.1) = 3.53, *p*<0.001, d = 0.97).

**Table 3:** Error analyses: Results of the 2 (SOA)×3 (congruency) ANOVAs for each group comparison.

Group comparison	Main effects			Interactions			
	SOA	congruency	group	SOA* congruency	SOA* group	congruency * group	SOA * congruency * group
monolinguals	*F*(1,306) = 3.32	*F*(2,306) = 13.79	*F*(1,306) = 5.10	*F*(2,306) = 1.51	*F*(1,306) = 1.22	*F*(2,306) = 1.03	*F*(2,306) = 1.31
vs. bilingual	*p* = 0.07	*p*<0.0001	*p*<0.05	*p* = 0.22	*p* = 0.27	*p* = 0.34	*p* = 0.27
L1	η^2^ = 0.009	η^2^ = 0.08	η^2^ = 0.01				
monolinguals	*F*(1,306) = 3.64	*F*(2,306) = 18.50	*F*(1,306) = 13.67	*F*(2,306)<1	*F*(1,306) = 2.12	*F*(2,306)<1	*F*(2,306) = 3.52
vs. bilingual	*p* = 0.06	*p*<0.0001	*p*<0.001		*p* = 0.15		*p*<0.05
L2	η^2^ = 0.01	η^2^ = 0.10	η^2^ = 0.04				η^2^ = 0.02
bilingual L1	*F*(1,24)<1	*F*(2,48) = 19.08	*F*(1,24) = 1.07	*F*(2,48) = 1.14	*F*(1,24)<1	*F*(2,48)<1	*F*(2,48) = 1.81
vs. L2		*p*<0.0001	*p* = 0.31	*p* = 0.33			*p* = 0.18
		η^2^ = 0.23					

### ERP results

#### Monolinguals

**Figure 4:**
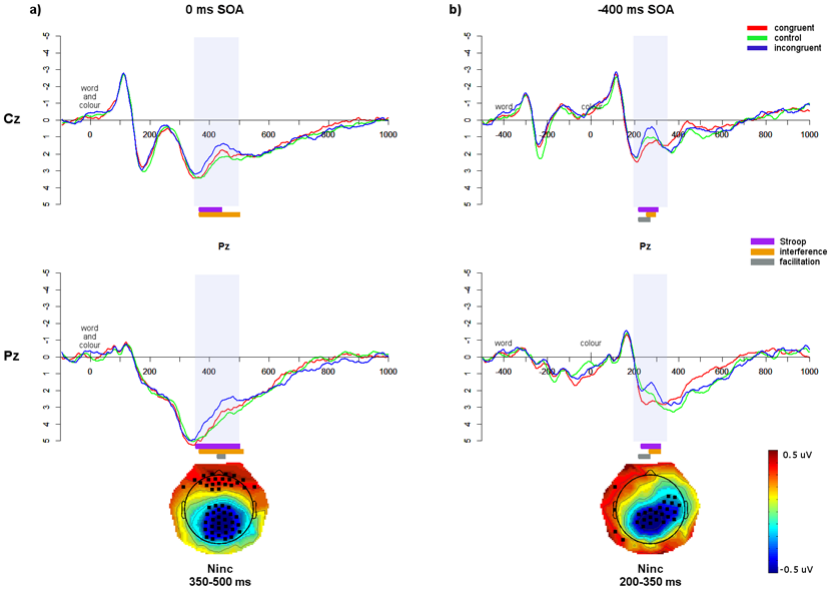
Monolingual ERP data. Monolingual English ERP waveforms at Cz and Pz for the a) 0 ms SOA; and b) −400 ms SOA. Significant effects from the running *t*-tests, within the N_inc_ windows (shaded area), are indicated in bars underneath. Topographic maps show the N_inc_ components (incongruent vs. congruent), with black dots indicating electrodes that show significant differences (*p*<0.05) between the average amplitudes of incongruent and congruent trials across the specified window.

In the 0 ms SOA, an N_inc_ occurred from approximately 350–500 ms (Figure 4), with significant Stroop and interference effects at Cz and Pz. As this SOA is analogous to the traditional Stroop task, this replicates previous ERP results [52]–[55], [59], [60], [90]. In the −400 ms SOA, an N_inc_ occurred from approximately 200–350 ms after colour presentation (Figure 4), with significant Stroop and interference effects at Cz and Pz. N_inc_ latencies were compared between SOAs using independent-samples *t*-tests. There was a significant difference between SOAs (*t*(27) = 17.92, *p*<0.0001, d = 3.40) such that the N_inc_ occurred earlier for the −400 ms SOA (258 ms), than for the 0 ms SOA (434 ms; Table 4).

**Table 4:** Summary of the N_inc_ windows, including peak latencies in the difference waves (averaged over Cz and Pz) for each group and SOA.

SOA	Group	Language	N_inc_ window	N_inc_ peak
0 ms	Monolinguals	English	350–500	434
SOA	Bilingual L1	Chinese	350–550	459
	Bilingual L2	English	400–600	529
−400 ms	Monolinguals	English	200–350	258
SOA	Bilingual L1	Chinese	200–350	299
	Bilingual L2	English	200–350	278

#### Bilinguals L1 Chinese

**Figure 5:**
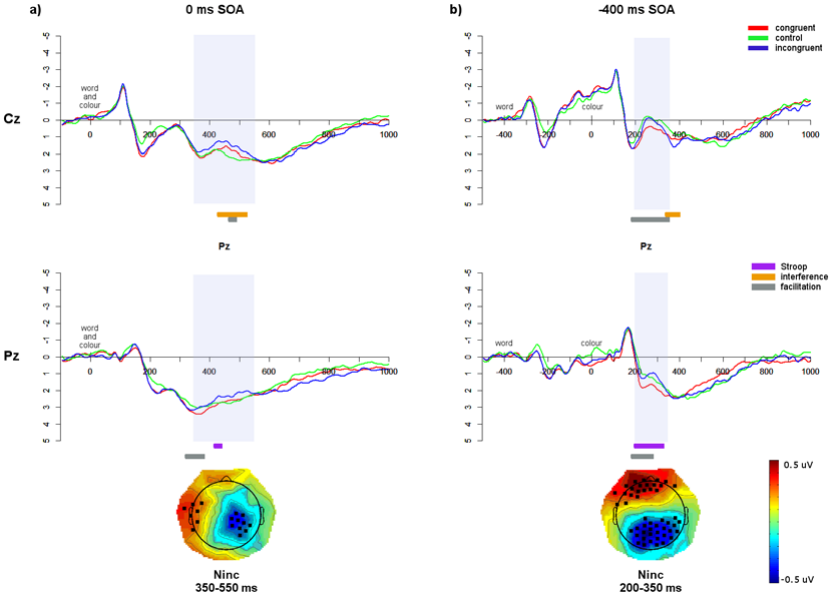
Bilingual L1 ERP data. Bilinguals L1 Chinese waveforms at Cz and Pz for the a) 0 ms SOA; and b) −400 ms SOA. Significant effects from the running *t*-tests, within the N_inc_ windows (shaded area), are indicated in bars underneath. Topographic maps show the N_inc_ components, plus electrodes showing significant differences (*p*<0.05) between incongruent and congruent trials across the specified window.

In the bilinguals L1 Chinese 0 ms SOA (Figure 5a), an N_inc_ occurred from approximately 350–550 ms, showing significant interference at Cz and a significant Stroop effect at Pz. In the −400 ms SOA, an N_inc_ occurred from approximately 200–350 ms after colour presentation, showing interference at Cz and Stroop effects at Pz (Figure 5b). Comparing component latencies, the N_inc_ showed a significantly earlier N_inc_ for the −400 ms SOA (299 ms) than for the 0 ms SOA (459 ms; *t*(24) = 10.67, *p*<0.0001, d = 2.21; Table 4).

#### Bilinguals L2 English

**Figure 6:**
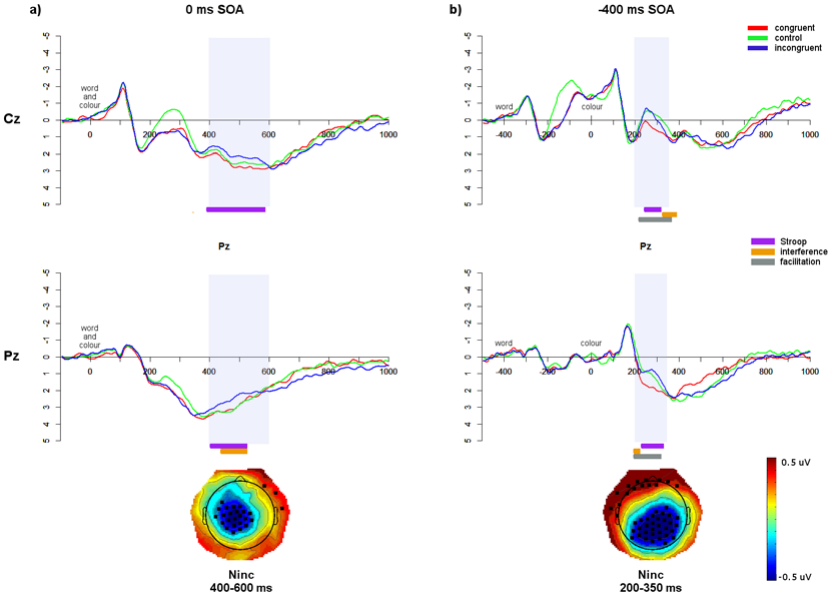
Bilingual L2 ERP data. Bilinguals L2 English waveforms at Cz and Pz for the a) 0 ms SOA; and b) −400 ms SOA. Significant effects from the running *t*-tests, within the N_inc_ windows (shaded area), are indicated in bars underneath. Topographic maps show the N_inc_ components, plus electrodes showing significant differences (*p*<0.05) between incongruent and congruent trials across the specified window.

For the bilingual L2 English 0 ms SOA (Figure 6a), an N_inc_ occurred from approximately 400–600 ms, with significant Stroop effects at Cz and Pz and significant interference effects at Pz. In the −400 ms SOA, an N_inc_ occurred from 200–350 ms after colour presentation, with significant Stroop and interference effects at Cz and Pz (Figure 6b). Comparing the latencies between SOAs, the N_inc_ occurred earlier in the −400 ms SOA (278 ms) than the 0 ms SOA (529 ms; *t*(24) = 15.10, *p*<0.0001, d = 3.03; Table 4).

#### Comparison of Stroop conflict effects between groups

**Figure 7:**
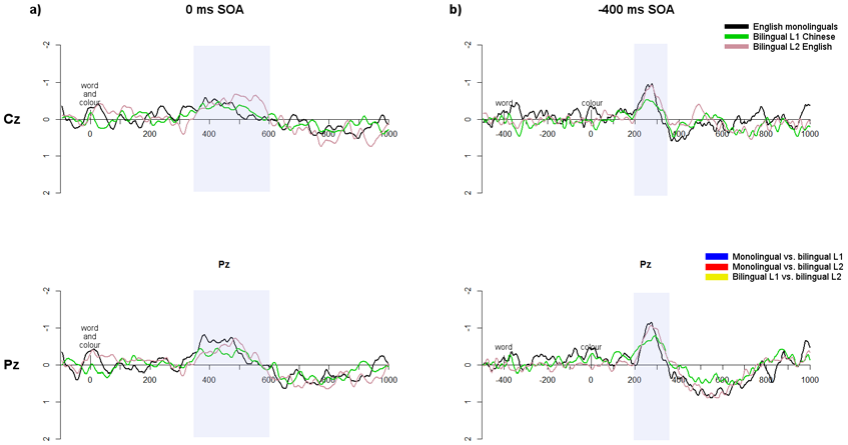
ERP difference waves. Difference waves (incongruent minus congruent) at Cz and Pz for each group in the a) 0 ms; and b) −400 ms SOAs. Significant differences between the groups, as evaluated by running *t*-tests, are plotted in bars below. Shaded regions show the approximate N_inc_ windows for each SOA.

Differences in conflict processing between groups were evaluated in the ERP data by comparing the amplitude of the difference waves (incongruent minus congruent) at the N_inc_ window using running *t*-tests. In the 0 ms SOA (Figure 7a), the N_inc_ onset occurred for all groups at approximately 400 ms. The negativity was more sustained for the bilingual L2, although there were no statistically significant amplitude differences in the running *t*-tests.

Because running *t*-tests compare the amplitude at each time point, they do not account for temporal shifts; therefore additional amplitude comparisons were performed by comparing the peak difference wave amplitude (averaged over Cz and Pz) within the specific N_inc_ windows for each group/language (Table 4) using *t*-tests. The bilingual L1 showed a slightly more negative N_inc_ amplitude than the monolinguals in the 0****ms SOA (monolinguals: **−**0.54 uV, *SE* = 0.13; bilinguals: **−**1.09, *SE* = 0.23; *t*(39.1) = 2.07, *p*<0.05, d = 57), although this difference does not come out in the group difference waves presented in Figure 7. There were no differences between monolinguals and the L2 or between the L1 and L2 at the 0****ms SOA (all *p*’s>0.11). There were no differences between any of the groups in the **−**400****ms SOA (all *p*’s>0.13).

To investigate differences in N_inc_ peak latency between the groups, latency analyses were performed on the difference waves for each SOA using the SOA- and group-specific N_inc_ windows (Table 4). The N_inc_ latencies were averaged over Cz and Pz and compared between groups with *t*-tests. In the 0 ms SOA N_inc_, the bilingual L2 N_inc_ peak (529 ms) occurred significantly later than both the L1 (459 ms, *t*(24) = 3.47, *p*<0.01, d = 0.70) and monolinguals (434 ms; *t*(44.6) = 5.89, *p*<0.0001, d = 1.62), but monolinguals and the L1 did not differ (*t*(40.9) = 1.43, *p* = 0.16, d = 0.39). This later peak is likely due to the more sustained N_inc_ component; note that in the difference waves, the N_inc_ onset was similar for all groups. In the −400 ms SOA, there was a slightly earlier N_inc_ for monolinguals (258 ms) than for the bilingual L1 (299 ms; *t*(50.7) = 2.77, *p*<0.01, d = 0.76) but no difference between monolinguals and L2 (278 ms; *t*(49.5) = 1.31, *p* = 0.20, d = 0.36) or between L1 and L2 (*t*(24) = 1.37, *p* = 0.18, d = 0.28). Therefore the N_inc_ in the −400 ms SOA occurred later for bilinguals than monolinguals.

#### Comparison of non-conflict-specific effects between groups

**Figure 8:**
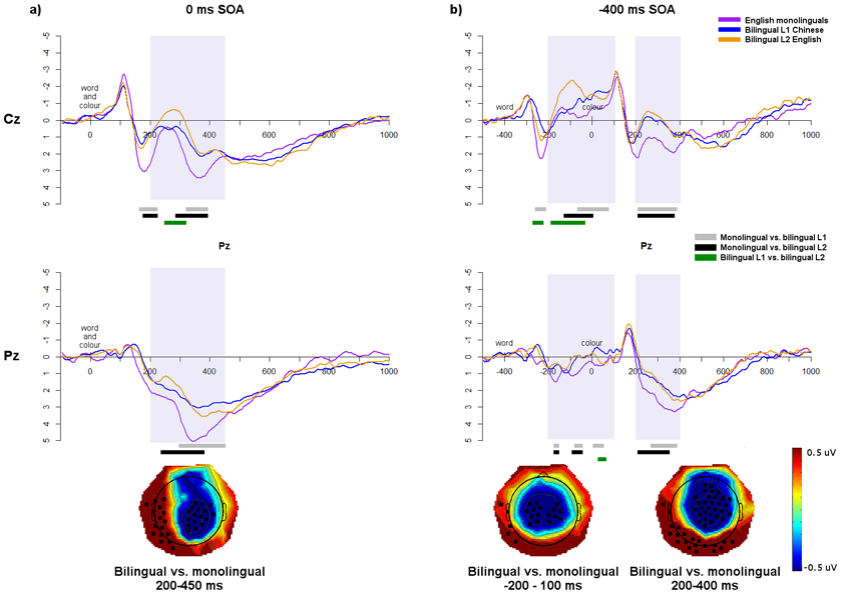
Comparison of ERP waveforms for control trials. Waveforms of control trials at Cz and Pz for each group in the a) 0 ms SOA; and b) −400 ms SOA. Significant differences between the groups, based on running *t*-tests, are indicated in bars underneath. Shaded regions indicate the windows of significant group differences, and topographic maps show the bilingual – monolingual differences (bilinguals averaged over L1 and L2) averaged across these windows; electrodes showing statistically significant differences (*p*<0.05) between the bilinguals and monolinguals across the specified window are indicated by a black square.

As in the behavioural data, the control conditions (‘%%%%’ in English, ‘%’ in Chinese) were compared in the ERP data to evaluate the impact of the non-conflict-specific effect. Running *t*-tests were performed from 200 ms after the first stimulus until 600 ms after the second stimulus in all SOAs, to restrict analyses to the time windows of interest (see Figure 8). In the 0 ms SOA, monolingual waveforms were significantly more positive than both the L1 and L2 from approximately 200–450 ms at Cz and Pz. Follow-up *t*-tests comparing the average amplitude over this window at Cz and Pz confirmed that monolinguals showed a more positive waveform than the bilingual L1 at Pz (*t*(50.2) = 3.09, *p*<0.001, d = 0.85) with a trend at Cz (*t*(50.6) = 1.94, *p* = 0.06, d = 0.53). The monolingual waveforms were also more positive than the bilingual L2 waveforms at Cz (*t*(48.2) = 2.64, *p*<0.05, d = 0.73) and Pz (*t*(49.4) = 2.62, *p*<0.05, d = 0.72). There were no differences between the L1 and L2 at either electrode (all *p*’s>0.16). As can be seen in the topoplots presented in Figure 8a, these effects were not limited to Cz and Pz but extended to a centro-parietal distribution.

In the −400 ms SOA, in the N_inc_ window from 200–400 ms, monolinguals had significantly more positive waveforms than the bilingual L1 at Cz (*t*(51) = 3.59, *p*<0.001, d = 0.99) and Pz (*t*(50.3) = 2.60, *p*<0.05, d = 0.72), and also more positive waveforms than the L2 at Cz (*t*(50.8) = 3.96, *p*<0.001, d = 1.09) and Pz (*t*(50.4) = 2.41, *p*<0.05, d = 0.66); there were no differences between the bilingual L1 and L2 at either electrode (all *p*’s>0.32). There were also group differences in an earlier window from approximately −200 to 100 ms (200–500 ms after word presentation). Averaging over this window, there was a trend of a difference between monolinguals at Pz (*t*(50.8) = 1.96, *p* = 0.06, d = 0.54), and a significant difference between monolinguals and L2 at Cz (*t*(48.3) = 2.92, *p*<0.01, d = 0.80) with a trend at Pz (*t*(50.8) = 1.66, *p* = 0.10, d = 0.46). The L2 was significantly more negative than the L1 in this window at Cz (*t*(24) = 2.50, *p*<0.05, d = 0.52) but not at Pz (*t*(24) = 0.38, *p* = 0.71). As can be seen in the topoplots presented in Figure 8b, these effects extended beyond Cz and Pz to a centralized scalp distribution.

## Discussion

The current study investigated the electrophysiological correlates of the bilingual advantage using a Stroop task with SOA manipulation. Two theories of bilingual executive control were contrasted: the BICA and the BEPA hypotheses. A conflict-specific bilingual advantage in inhibitory control, as predicted by the BICA hypothesis, was evaluated by comparing Stroop and interference effects in the behavioural data, and the N_inc_ component in the ERP data. A non-conflict-specific bilingual advantage in more domain-general executive control, as predicted by the BEPA hypothesis, was evaluated by comparing the non-linguistic, non-semantic control trials in both the behavioural and ERP data. Each hypothesis will be evaluated in turn.

### The conflict-specific bilingual advantage: evaluation of the BICA hypothesis

The BICA hypothesis predicted a bilingual advantage in conflict processing such that bilinguals would demonstrate smaller behavioural interference effects and smaller N_inc_ amplitudes [64], [91] compared to monolinguals. Behaviourally, when comparing monolinguals to the bilingual L1, there were no significant differences in conflict effects in any SOA. However, bilinguals showed smaller interference effects in their L2 than monolinguals in the 0 ms SOA. These L2 results ostensibly support the BICA hypothesis; however, as mentioned in the Introduction, the decreased interference in the L2 could instead be a result of reduced language proficiency rather than enhanced cognitive control [43]. In a weaker language, word frequencies in L2 are lower than in the L1 (see the temporal delay hypothesis of the BIA+ model [22]) and the conceptual links between words and concepts are not as strong (see the weaker links hypothesis [92]). This could conceptually lead to smaller interference effects not because of enhanced cognitive control, but because of a smaller influence of the distracting word. A conflict-specific bilingual advantage would therefore also require a significant effect in the L1 compared to monolinguals. However, there were no significant differences in conflict effects when comparing monolinguals to the bilingual L1 in any SOA. Thus we did not find evidence for a behavioural conflict-specific bilingual advantage.

In the ERP data, conflict processing was evaluated via the N_inc_ ERP component. When comparing N_inc_ peaks there was a slightly more negative N_inc_ amplitude in the 0 ms SOA for the bilingual L1 compared to monolinguals; however, this difference was not reflected when comparing the groups with running *t*-tests or in the difference waves plotted in Figure 7. Furthermore, there were no differences in N_inc_ amplitude when comparing monolinguals to the L2. As discussed above, because differences did not occur in both languages, this does not support the BICA hypothesis.

Interestingly, the bilingual L2 showed a more sustained N_inc_ in the 0 ms and −400 ms SOAs compared to the L1 and monolinguals, although there were no amplitude differences. One potential explanation for this more sustained component is that activating the semantic information of the word may be more effortful in the L2 due to its reduced automaticity or weaker links [22], [87], leading to difficulties in evaluating the presence of conflict.

Previous research has demonstrated that bilinguals experience significant delays in lexical processing, both in the L1 and L2 ([93]–[100]; see [24], [101], [102] for reviews). Specifically, we have recently observed a delay of 100 ms in early orthographic recognition in a second language (Coderre, 2012, unpublished doctoral dissertation). Because Stroop conflict originates from the incongruent information of the word stimulus, it is heavily dependent on the speed of lexico-semantic access: the word must first be activated in order to detect or resolve conflict. Thus a delay in language processing in the less-proficient language may also lead to a delay in the onset of conflict processing in the Stroop task, leading to a delayed N_inc_ in the L2 compared to the L1 or to monolinguals.

Interestingly, however, despite the more sustained N_inc_ in the bilingual L2, all groups showed a similar negativity *onset* at the N_inc_ window, suggesting that conflict processing was initiated at the same latency in all groups. Using this same dataset we have observed a significant delay of the N170 component, an early ERP indexing orthographic processing, in the bilingual L2 of 100 ms (Coderre, 2012, unpublished doctoral dissertation). Thus despite a substantial delay in lexical access speed in the L2, the onset of conflict processing was not delayed in the L2 in the current data. This suggests that early linguistic processing delays do not persist across all subsequent processing, but catch up at some point along the way. It may be that conflict detection processes do not wait until full semantic access of the word is achieved, and that even partial semantic activation is enough to trigger conflict detection processes. There are likely a number of other cognitive functions occurring between lexical access and conflict detection which may have contributed to this alleged compensation in the L2 processing delay. Where exactly this compensation would occur requires further research; however, the interaction of conflict processing and language processing speed is an interesting avenue for future research.

Interestingly, the bilingual L1 experienced a significantly later N_inc_ in the −400 ms SOA compared to monolinguals. This delayed onset of conflict processing in bilinguals could be indicative of enhanced inhibitory control over the L1. As language was blocked in this paradigm, bilinguals may have exerted more control over their L1 (as the stronger language) to avoid interference throughout the entire block, slowing conflict detection processes and leading to later N_inc_ components. In the 0 ms SOA, being the more cognitively demanding condition, cognitive control may also have been heightened in monolinguals, creating a similar effect and equalizing conflict processing speed between groups, which would explain the lack of group differences in N_inc_ latencies. This is a tentative interpretation, as no differences occurred in behavioural interference effects or N_inc_ amplitudes; bilinguals could alternatively have an enhanced ability to ignore L1 word meanings for the task at hand (see next section). Nevertheless, these differences in N_inc_ latencies at long-latency SOAs may be an interesting way of assessing language control.

In sum, the evidence for a conflict-specific bilingual advantage supporting the BICA hypothesis is limited in the current data: although group differences occurred in interference effects and the N_inc_ component, these differences were not consistent across languages. The tenuous expression of a conflict-specific advantage in the current data supports Hilchey & Klein’s [9] conclusion that the bilingual advantage in conflict processing is a sporadic and elusive phenomenon. We next turn our attention to evaluations of the BEPA hypothesis.

### The non-conflict-specific bilingual advantage: evaluation of the BEPA hypothesis

A non-conflict-specific bilingual advantage in domain-general executive control (BEPA hypothesis) was investigated by comparing control trials, both behaviourally and in the EEG data. Bilinguals experienced a behavioural non-conflict-specific advantage in both languages, such that they showed faster control RTs than monolinguals in the non-linguistic, non-conflict control trials. This advantage occurred in both languages for the bilinguals. Importantly, this was not a speed-accuracy trade-off, as error analyses indicated that monolinguals made more errors overall than bilinguals, as well as being slower overall in the control condition. Thus overall, the behavioural data support the BEPA hypothesis, demonstrating a general enhancement in executive control that is not affected by language context.

In the ERP data, comparisons of control trials across groups yielded significant differences in all SOAs. Specifically, bilinguals showed more negative waveforms within the N_inc_ windows, in both languages, than monolinguals. Because this enhanced negativity in the non-linguistic, non-conflict control trials was associated with faster behavioural RTs, this reflects more efficient cognitive processing in bilinguals, as predicted by the BEPA hypothesis. Additionally, these findings go against the BICA hypothesis, which would predict no differences between groups in the absence of conflict.

Crucially, in the −400 ms SOA the increased negativity for bilinguals occurred not only at the N_inc_ window but also from −200 to 100 ms before colour presentation (200–500 ms after control stimulus presentation). In the −400 ms SOA, upon word presentation it is not yet known whether the stimulus will be congruent or incongruent, as the colour has not appeared yet. However, we observed differences in the EEG waveforms between monolinguals and bilinguals *before* the onset of the colour, and therefore before the onset of conflict. Such early differences suggest that the bilingual executive processing advantage does not arise from more efficient conflict monitoring processes [33] but from more domain-general cognitive processing. This suggests that bilinguals were handling the pre-exposed distractor stimulus differently from monolinguals. We propose that bilinguals may be more efficiently engaging a control mechanism such as suppression of distracting or irrelevant information, regardless of the presence of conflict or semantic salience.

In support of this conjecture, we have recently demonstrated, using this Stroop SOA paradigm with fMRI in monolinguals [103], that relative to simultaneous stimuli presentation in the 0 ms SOA, negative SOAs elicited greater task-related signal change in the left inferior frontal gyrus (LIFG), a region believed to implement cognitive control via suppression of irrelevant semantic information [26], [104]–[106]. Because this effect was greater in response to the pre-exposed word in negative SOAs, it was hypothesized that these monolingual participants were engaging a proactive strategy of ‘distractor suppression’ in order to avoid conflict when the colour stimulus was subsequently presented. This mechanism recalls the dual mechanisms of control theory of Braver and colleagues [107]–[109], which proposes two mechanisms of cognitive control: one a ‘late correction’ reactive response engaged to resolve conflict once it has occurred; and one a proactive ‘early selection’ strategy engaged to pre-emptively reduce control demands for when conflict occurs. The fact that group differences were observed before the onset of conflict in the current ERP data may suggest that this mechanism of distractor suppression, while engaged by all participants, is more efficient in bilinguals. This is in line with recent evidence from Morales et al. [110] who, using a continuous performance task requiring both proactive monitoring and reactive inhibition, found that bilinguals were more efficient at adjusting between proactive and reactive control. Thus a mechanism of proactive distractor suppression could explain the more negative ERP amplitudes and correspondingly faster behavioural control RTs observed in bilinguals in the current data.

As a caveat, it should be mentioned that based on the current data and literature, it is unclear whether this mechanism underlying the non-conflict-specific bilingual advantage deals with distracting and/or irrelevant stimuli via active inhibition of the information, as proposed here as a difference in ‘distractor suppression’, or via a more passive ignoring of the information by top-down guidance of attention, as proposed by Hernández et al. [49]. These are two subtly different aspects of executive control (e.g. see [111]) that the present paradigm was not designed to distinguish between. To some extent this is two sides of the same coin, but future research should seek to make this distinction by designing studies specifically aimed at teasing apart attentional control vs. distractor suppression influences in bilinguals. For example, we are pursuing the possibility that an enhanced ability of bilinguals to direct attention to the target stimulus or away from the irrelevant stimulus would be manifest in early visual components like the N1 [112]. In any case, the crucial finding in this study is that group differences in executive control performance were observed in control trials in the current paradigm, which contained no conflict or semantic information, and occurred before the onset of conflict in the −400 ms SOA. We propose that this provides evidence of a domain-general mechanism of proactive distractor suppression and/or top-down attentional guidance which operates independently of both language context and the presence of conflict, as predicted by the BEPA hypothesis. Therefore this study adds to the extant literature demonstrating that the bilingual advantage is not specific to inhibitory control but extends to more global executive processing (e.g. [9], [33], [49], [110]).

Additional factors may have affected the results obtained here. It is still unclear how language-specific characteristics of a bilingual population, including variables like L2 proficiency, age of acquisition, and immersion experience influence the presence and magnitude of the bilingual advantage. For example, although the bilinguals tested here were relatively low-proficiency, they were all immersed in an L2 environment at the time of testing, which may lead to differences when comparing to other studies. Future studies should also replicate these results with bilinguals of different languages, to address whether native languages other than Chinese show similar effects as those described here. It is also possible that other factors besides bilingual status could be contributing to the observed group differences, such as intelligence, verbal abilities, or working memory. Although we did not investigate these variables in the current study, other studies controlling for factors like intelligence, video game experience, and digit span have also reported a bilingual advantage (e.g. [30],[32],[49]), so we feel confident that these effects are primarily driven by bilingual status. Nevertheless, these are important factors to keep in mind for future research.

The current findings should also be replicated using a non-linguistic conflict task. Although we compared the non-linguistic control trials in an attempt to eliminate linguistic influences when evaluating the bilingual advantage, it is possible that bilinguals may have more efficient mechanisms of distractor suppression in a language context. Future studies should test for more efficient mechanisms of distractor suppression in non-linguistic tasks, for example in a flanker task with varying SOAs.

In conclusion, the current data demonstrated some significant, but inconsistent, evidence for a conflict-specific bilingual advantage. However, there was evidence for a non-conflict-specific advantage in bilinguals. Specifically, the ERP data demonstrated group differences before the onset of conflict, suggesting that bilinguals may exhibit more efficient mechanisms for the management of irrelevant stimuli, regardless of the presence of conflict or semantic information. Being one of only a few studies to investigate the bilingual advantage using EEG, this work sheds a new light on this phenomenon and demonstrates that the advantage is a result of a proactive management of the environment, rather than a reactive response to it.

## References

[1] Stroop J (1935) Studies of interference in serial verbal reactions. J Exp Psychol Gen 18: 643–662.

[2] MacLeod CM (1991) Half a century of research on the Stroop effect: an integrative review. Psychol Bull 109: 163–203. 2034749 10.1037/0033-2909.109.2.163

[3] Unsworth N, Engle RW (2007) The nature of individual differences in working memory capacity: Active maintenance in primary memory and controlled search from secondary memory. Psychol Rev 114: 104–132. 17227183 10.1037/0033-295X.114.1.104

[4] Gray JR, Chabris CF, Braver TS (2003) Neural mechanisms of general fluid intelligence. Nat Neurosci 6: 316–322. 12592404 10.1038/nn1014

[5] Bialystok E (2009) Bilingualism: The good, the bad, and the indifferent. Biling Lang Cogn 12: 3–11.

[6] Bialystok E, Craik FIM, Green DW, Gollan TH (2009) Bilingual Minds. Psychol Sci Public Interes 10: 89–129. 10.1177/1529100610387084 26168404

[7] Bialystok E (2011) Reshaping the mind: The benefits of bilingualism. Can J Exp Psychol 65: 229–235. 21910523 10.1037/a0025406 PMC4341987

[8] Tao L, Marzecová A, Taft M, Asanowicz D, Wodniecka Z (2011) The efficiency of attentional networks in early and late bilinguals: the role of age of acquisition. Front Psychol 2: 1–19. 21713011 10.3389/fpsyg.2011.00123 PMC3114252

[9] Hilchey MD, Klein RM (2011) Are there bilingual advantages on nonlinguistic interference tasks? Implications for the plasticity of executive control processes. Psychon Bull Rev 18: 625–658. 21674283 10.3758/s13423-011-0116-7

[10] van Hell JG, Dijkstra T (2002) Foreign language knowledge can influence native language performance in exclusively native contexts. Psychon Bull Rev 9: 780–789. 12613683 10.3758/bf03196335

[11] Degani T, Tokowicz N (2010) Semantic ambiguity within and across languages: an integrative review. Q J Exp Psychol 63: 1266–1303. 10.1080/17470210903377372 19953429

[12] Poulisse N, Bongaerts T (1994) First language use in second language production. Appl Linguist 15: 36–57.

[13] Soares C, Grosjean F (1984) Bilinguals in a monolingual and a bilingual speech mode: The effect on lexical access. Mem Cognit 12: 380–386. 10.3758/bf03198298 6503701

[14] Martin CD, Dering B, Thomas EM, Thierry G (2009) Brain potentials reveal semantic priming in both the “active” and the “non-attended” language of early bilinguals. Neuroimage 47: 326–333. 19374949 10.1016/j.neuroimage.2009.04.025

[15] Midgley K, Holcomb P, van Heuven WJB, Grainger J (2008) An electrophysiological investigation of cross-language effects of orthographic neighborhood. Brain Res 1246: 123–135. 18948089 10.1016/j.brainres.2008.09.078 PMC2656968

[16] Rodriguez-Fornells A, van der Lugt A, Rotte M, Britti B, Heinze H-J, et al (2005) Second language interferes with word production in fluent bilinguals: brain potential and functional imaging evidence. J Cogn Neurosci 17: 422–433. 15814002 10.1162/0898929053279559

[17] van Heuven WJB, Schriefers H, Dijkstra T, Hagoort P (2008) Language conflict in the bilingual brain. Cereb Cortex 18: 2706–2716. 18424776 10.1093/cercor/bhn030 PMC2567421

[18] Rodriguez-Fornells A, Rotte M, Heinze H-J, Nösselt T, Münte TF (2002) Brain potential and functional MRI evidence for how to handle two languages with one brain. Nature 415: 1026–1029. 11875570 10.1038/4151026a

[19] Brysbaert M, Duyck W (2010) Is it time to leave behind the Revised Hierarchical Model of bilingual language processing after fifteen years of service? Biling Lang Cogn 13: 359–371.

[20] Kroll JF, Dussias PE, Bogulski CA, Valdes KroffJR (2012) Juggling Two Languages in One Mind: What Bilinguals Tell Us About Language Processing and its Consequences for Cognition. In: Ross BH, editor. The Psychology of Learning and Motivation. USA: Elsevier, Vol. 56: 229–262.

[21] Kroll JF, Bobb SC, Wodniecka Z (2006) Language selectivity is the exception, not the rule: Arguments against a fixed locus of language selection in bilingual speech. Biling Lang Cogn 9: 119–135.

[22] Dijkstra T, van Heuven WJB (2002) The architecture of the bilingual word recognition system: From identification to decision. Biling Lang Cogn 5: 175–197.

[23] Green DW (1998) Mental control of the bilingual lexico-semantic system. Biling Lang Cogn 1: 67–81.

[24] van Heuven WJB, Dijkstra T (2010) Language comprehension in the bilingual brain: fMRI and ERP support for psycholinguistic models. Brain Res Rev 64: 104–122. 20227440 10.1016/j.brainresrev.2010.03.002

[25] Hernandez AE, Meschyan G (2006) Executive function is necessary to enhance lexical processing in a less proficient L2: Evidence from fMRI during picture naming. Biling Lang Cogn 9: 177–188.

[26] Ye Z, Zhou X (2009) Conflict control during sentence comprehension: fMRI evidence. Neuroimage 48: 280–290. 19540923 10.1016/j.neuroimage.2009.06.032

[27] Costa A, Sebastián-Gallés N (2014) How does the bilingual experience sculpt the brain? Nat Rev Neurosci 15: 336–345. 24739788 10.1038/nrn3709 PMC4295724

[28] Garbin G, Sanjuan A, Forn C, Bustamante JC, Rodriguez-Pujadas A, et al (2010) Bridging language and attention: Brain basis of the impact of bilingualism on cognitive control. Neuroimage 53: 1272–1278. 20558314 10.1016/j.neuroimage.2010.05.078

[29] Prior A, Macwhinney B (2010) A bilingual advantage in task switching. Biling Lang Cogn 13: 253–262. 10.1017/S1366728909990526 PMC9724810 36479004

[30] Bialystok E, Martin MM (2004) Attention and inhibition in bilingual children: evidence from the dimensional change card sort task. Dev Sci 7: 325–339. 15595373 10.1111/j.1467-7687.2004.00351.x

[31] Bialystok E, Craik FIM, Ruocco AC (2006) Dual-modality monitoring in a classification task: the effects of bilingualism and ageing. Q J Exp Psychol 59: 1968–1983. 10.1080/17470210500482955 16987784

[32] Bialystok E, Shapero D (2005) Ambiguous benefits: The effect of bilingualism on reversing ambiguous figures. Dev Sci 8: 595–604. 16246250 10.1111/j.1467-7687.2005.00451.x

[33] Costa A, Hernández M, Costa-Faidella J, Sebastián-Gallés N (2009) On the bilingual advantage in conflict processing: Now you see it, now you don’t. Cognition 113: 135–149. 19729156 10.1016/j.cognition.2009.08.001

[34] Carlson SM, Meltzoff AN (2008) Bilingual experience and executive functioning in young children. Dev Sci 11: 282–298. 18333982 10.1111/j.1467-7687.2008.00675.x PMC3647884

[35] Goetz PJ (2003) The effects of bilingualism on theory of mind development. Biling Lang Cogn 6: 1–15.

[36] Bialystok E, Senman L (2004) Executive processes in appearance-reality tasks: The role of inhibition of attention and symbolic representation. Child Dev 75: 562–579. 15056206 10.1111/j.1467-8624.2004.00693.x

[37] Bartolotti J, Marian V, Schroeder SR, Shook A (2011) Bilingualism and inhibitory control influence statistical learning of novel word forms. Front Psychol 2: 1–10. 22131981 10.3389/fpsyg.2011.00324 PMC3223905

[38] Bialystok E (2006) Effect of bilingualism and computer video game experience on the Simon task. Can J Exp Psychol 60: 68–79. 16615719 10.1037/cjep2006008

[39] Bialystok E, Craik FIM, Klein R, Viswanathan M (2004) Bilingualism, aging, and cognitive control: evidence from the Simon task. Psychol Aging 19: 290–303. 15222822 10.1037/0882-7974.19.2.290

[40] Bialystok E, Craik FIM, Luk G (2008) Cognitive control and lexical access in younger and older bilinguals. J Exp Psychol Learn Mem Cogn 34: 859–873. 18605874 10.1037/0278-7393.34.4.859

[41] Bialystok E, Depape A-M (2009) Musical expertise, bilingualism, and executive functioning. J Exp Psychol Hum Percept Perform 35: 565–574. 19331508 10.1037/a0012735

[42] Martin-Rhee MM, Bialystok E (2008) The development of two types of inhibitory control in monolingual and bilingual children. Biling Lang Cogn 11: 81–93.

[43] Coderre EL, van Heuven WJB, Conklin K (2013) The timing and magnitude of Stroop interference and facilitation in monolinguals and bilinguals. Biling Lang Cogn 16: 420–441. 10.1017/S1366728912000405 PMC3590568 23483406

[44] Singh N, Mishra RK (2013) Second language proficiency modulates conflict-monitoring in an oculomotor Stroop task: evidence from Hindi-English bilinguals. Front Psychol 4: 1–10. 23781210 10.3389/fpsyg.2013.00322 PMC3679481

[45] Singh N, Mishra RK (2012) Does language proficiency modulate oculomotor control? Evidence from Hindi–English bilinguals. Biling Lang Cogn 15: 771–781.

[46] Paap KR, Greenberg ZI (2013) There is no coherent evidence for a bilingual advantage in executive processing. Cogn Psychol 66: 232–258. 23370226 10.1016/j.cogpsych.2012.12.002

[47] Duñabeitia JA, Hernández JA, Antón E, Macizo P, Estévez A, et al (2013) The inhibitory advantage in bilingual children revisited: myth or reality? Exp Psychol: 1–18. doi:10.1027/1618-3169/a000243 10.1027/1618-3169/a000243 24217139

[48] Bialystok E, Craik FIM, Grady C, Chau W, Ishii R, et al (2005) Effect of bilingualism on cognitive control in the Simon task: evidence from MEG. Neuroimage 24: 40–49. 15588595 10.1016/j.neuroimage.2004.09.044

[49] Hernández M, Costa A, Humphreys GW (2012) Escaping capture: bilingualism modulates distraction from working memory. Cognition 122: 37–50. 21890125 10.1016/j.cognition.2011.08.002

[50] Luk G, Anderson JAE, Craik FIM, Grady C, Bialystok E (2010) Distinct neural correlates for two types of inhibition in bilinguals: Response inhibition versus interference suppression. Brain Cogn 74: 347–357. 20965635 10.1016/j.bandc.2010.09.004

[51] Abutalebi J, Della Rosa PA, Green DW, Hernandez M, Scifo P, et al (2012) Bilingualism Tunes the Anterior Cingulate Cortex for Conflict Monitoring. Cereb Cortex 22: 2076–2086. 22038906 10.1093/cercor/bhr287

[52] Appelbaum LG, Meyerhoff KL, Woldorff MG (2009) Priming and backward influences in the human brain: Processing interactions during the Stroop interference effect. Cereb Cortex 19: 2508–2521. 19321654 10.1093/cercor/bhp036 PMC2764508

[53] Larson MJ, Kaufman DAS, Perlstein WM (2009) Neural time course of conflict adaptation effects on the Stroop task. Neuropsychologia 47: 663–670. 19071142 10.1016/j.neuropsychologia.2008.11.013

[54] Liotti M, Woldorff MG, Perez R, Mayberg HS (2000) An ERP study of the temporal course of the Stroop color-word interference effect. Neuropsychologia 38: 701–711. 10689046 10.1016/s0028-3932(99)00106-2

[55] Markela-Lerenc J, Ille N, Kaiser S, Fiedler P, Mundt C, et al (2004) Prefrontal-cingulate activation during executive control: Which comes first? Cogn Brain Res 18: 278–287. 10.1016/j.cogbrainres.2003.10.013 14741314

[56] West R (2003) Neural correlates of cognitive control and conflict detection in the Stroop and digit-location tasks. Neuropsychologia 41: 1122–1235. 12667546 10.1016/s0028-3932(02)00297-x

[57] Coderre EL, Conklin K, van Heuven WJB (2011) Electrophysiological measures of conflict detection and resolution in the Stroop task. Brain Res 1413: 51–59. 21840503 10.1016/j.brainres.2011.07.017

[58] Kutas M, Hillyard S (1980) Reading Senseless Sentences: Brain Potentials Reflect Semantic Incongruity. Science (80-) 207: 203–205. 10.1126/science.7350657 7350657

[59] Badzakova-Trajkov G, Barnett KJ, Waldie KE, Kirk IJ (2009) An ERP investigation of the Stroop task: the role of the cingulate in attentional allocation and conflict resolution. Brain Res 1253: 139–148. 19084509 10.1016/j.brainres.2008.11.069

[60] Hanslmayr S, Pastötter B, Bäuml K-H, Gruber S, Wimber M, et al (2008) The electrophysiological dynamics of interference during the Stroop task. J Cogn Neurosci 20: 215–225. 18275330 10.1162/jocn.2008.20020

[61] Mansouri FA, Tanaka K, Buckley MJ (2009) Conflict-induced behavioural adjustment: a clue to the executive functions of the prefrontal cortex. Nat Rev Neurosci 10: 141–152. 19153577 10.1038/nrn2538

[62] Ridderinkhof KR, Ullsperger M, Crone EA, Nieuwenhuis S (2004) The role of the medial frontal cortex in cognitive control. Science (80-) 306: 443–447. 10.1126/science.1100301 15486290

[63] Carter C, van Veen V (2007) Anterior cingulate cortex and conflict detection: an update of theory and data. Cogn Affect Behav Neurosci 7: 367–379. 18189010 10.3758/cabn.7.4.367

[64] Kousaie S, Phillips NA (2012) Conflict monitoring and resolution: Are two languages better than one? Evidence from reaction time and event-related brain potentials. Brain Res 1446: 71–90. 22356886 10.1016/j.brainres.2012.01.052

[65] Tillman CM, Wiens S (2011) Behavioral and ERP indices of response conflict in Stroop and flanker tasks. Psychophysiology 48: 1405–1411. 21457276 10.1111/j.1469-8986.2011.01203.x

[66] Bartholow BD, Pearson MA, Dickter CL, Sher KJ, Fabiani M, et al (2005) Strategic control and medial frontal negativity: Beyond errors and response conflict. Psychophysiology 42: 33–42. 15720579 10.1111/j.1469-8986.2005.00258.x

[67] Melara RD, Wang H, Vu K-PL, Proctor RW (2008) Attentional origins of the Simon effect: Behavioral and electrophysiological evidence. Brain Res 1215: 147–159. 18474363 10.1016/j.brainres.2008.03.026

[68] Swick D, Turken AU (2002) Dissociation between conflict detection and error monitoring in the human anterior cingulate cortex. Proc Natl Acad Sci 99: 16354–16359. 12456882 10.1073/pnas.252521499 PMC138615

[69] West R, Alain C (2000) Effects of task context and fluctuations of attention on neural activity supporting performance of the Stroop task. Brain Res 873: 102–111. 10915815 10.1016/s0006-8993(00)02530-0

[70] Holmes A, Pizzagalli DA (2008) Response conflict and frontocingulate dysfunction in unmedicated participants with major depression. Neuropsychologia 46: 2904–2913. 18577391 10.1016/j.neuropsychologia.2008.05.028 PMC2538441

[71] Dyer FN, Severance LJ (1973) Stroop interference with successive presentations of separate incongruent words and colors. J Exp Psychol 98: 438–439. 4705635 10.1037/h0034353

[72] Dyer FN (1971) The duration of word meaning responses: Stroop interference for different preexposures of the word. Psychon Sci 25: 229–231.

[73] Glaser MO, Glaser WR (1982) Time course analysis of the Stroop phenomenon. J Exp Psychol Hum Percept Perform 8: 875–894. 6218237 10.1037//0096-1523.8.6.875

[74] Glaser WR, Glaser MO (1989) Context effects in stroop-like word and picture processing. J Exp Psychol Gen 118: 13–42. 2522504 10.1037//0096-3445.118.1.13

[75] Appelbaum LG, Boehler C, Won R, Davis L, Woldorff MG (2012) Strategic Allocation of Attention Reduces Temporally Predictable Stimulus Conflict. J Cogn Neurosci 24: 1834–1848. 22360623 10.1162/jocn_a_00209 PMC3632454

[76] Pulvermüller F, Assadollahi R, Elbert T (2001) Neuromagnetic evidence for early semantic access in word recognition. Eur J Neurosci 13: 201–205. 11135019 10.1046/j.0953-816x.2000.01380.x

[77] Dell’Acqua R, Pesciarelli F, Jolicoeur P, Eimer M, Peressotti F (2007) The interdependence of spatial attention and lexical access as revealed by early asymmetries in occipito-parietal ERP activity. Psychophysiology 44: 436–443. 17371492 10.1111/j.1469-8986.2007.00514.x

[78] Bialystok E (1999) Cognitive complexity and attentional control in the bilingual mind. Child Dev 70: 636–644.

[79] Bialystok E (2010) Global-local and trail-making tasks by monolingual and bilingual children: Beyond inhibition. Dev Psychol 46: 93–105. 20053009 10.1037/a0015466 PMC2805165

[80] Costa A, Hernández M, Sebastián-Gallés N (2008) Bilingualism aids conflict resolution: evidence from the ANT task. Cognition 106: 59–86. 17275801 10.1016/j.cognition.2006.12.013

[81] Bialystok E, Craik FIM, Ryan J (2006) Executive control in a modified antisaccade task: Effects of aging and bilingualism. J Exp Psychol Learn Mem Cogn 32: 1341–1354. 17087588 10.1037/0278-7393.32.6.1341

[82] Mohamed Zied K, Phillipe A, Pinon K, Havet-Thomassin V, Aubin G, et al (2004) Bilingualism and adult differences in inhibitory mechanisms: evidence from a bilingual Stroop task. Brain Cogn 54: 254–256. 15050787 10.1016/j.bandc.2004.02.036

[83] Sumiya H, Healy AF (2004) Phonology in the bilingual Stroop effect. Mem Cognit 32: 752–758. 10.3758/bf03195865 15552352

[84] Braet W, Noppe N, Wagemans J, Op de Beeck H (2011) Increased Stroop interference with better second-language reading skill. Q J Exp Psychol 64: 596–607. 10.1080/17470218.2010.513735 20924988

[85] Naylor LJ, Stanley EM, Wicha NYY (2012) Cognitive and electrophysiological correlates of the bilingual Stroop effect. Front Psychol 3: 1–18. 22485099 10.3389/fpsyg.2012.00081 PMC3317261

[86] Chouiter L, Dieguez S, Annoni J-M, Spierer L (2014) High and low stimulus-driven conflict engage segregated brain networks, not quantitatively different resources. Brain Topogr 27: 279–292. 23813270 10.1007/s10548-013-0303-0

[87] Gollan TH, Montoya RI, Bonanni MP (2005) Proper names get stuck on bilingual and monolingual speakers’ tip of the tongue equally often. Neuropsychology 19: 278–287. 15910114 10.1037/0894-4105.19.3.278

[88] Janse E (2012) A non-auditory measure of interference predicts distraction by competing speech in older adults. Aging, Neuropsychol Cogn 19: 741–758. 10.1080/13825585.2011.652590 22293017

[89] Jesse A, Janse E (2012) Audiovisual benefit for recognition of speech presented with single-talker noise in older listeners. Lang Cogn Process 27: 1167–1191.

[90] West R, Alain C (1999) Event-related neural activity associated with the Stroop task. Cogn Brain Res 8: 157–164. 10.1016/s0926-6410(99)00017-8 10407204

[91] Heidlmayr K, Moutier S, Hemforth B, Isel F (2012) Bilingualism and executive functions: ERP evidence from a Stroop task. Cognitive Neuroscience Society.

[92] Gollan TH, Montoya RI, Fennema-Notestine C, Morris SK (2005) Bilingualism affects picture naming but not picture classification. Mem Cognit 33: 1220–1234. 10.3758/bf03193224 16532855

[93] Ardal S, Donald MW, Meuter R, Muldrew S, Luce M (1990) Brain responses to semantic incongruity in bilinguals. Brain Lang 39: 187–205. 2224493 10.1016/0093-934x(90)90011-5

[94] Elston-Güttler KE, Friederici AD (2005) Memory and Language Native and L2 processing of homonyms in sentential context. J Mem Lang 52: 256–283.

[95] Liu Y, Perfetti CA (2003) The time course of brain activity in reading English and Chinese: An ERP study of Chinese bilinguals. Hum Brain Mapp 18: 167–175. 12599274 10.1002/hbm.10090 PMC6871937

[96] Moreno EM, Kutas M (2005) Processing semantic anomalies in two languages: An electrophysiological exploration in both languages of Spanish-English bilinguals. Cogn Brain Res 22: 205–220. 10.1016/j.cogbrainres.2004.08.010 15653294

[97] Newman AJ, Tremblay A, Nichols E, Neville HJ, Ullman MT (2012) The Influence of Language Proficiency on Lexical Semantic Processing in Native and Late Learners of English. J Cogn Neurosci 24: 1205–1223. 21981676 10.1162/jocn_a_00143 PMC4447492

[98] Phillips NA, Segalowitz N, O’Brien I, Yamasaki N (2004) Semantic priming in a first and second language: evidence from reaction time variability and event-related brain potentials. J Neurolinguistics 17: 237–262.

[99] Phillips NA, Klein D, Mercier J, de Boysson C (2006) ERP measures of auditory word repetition and translation priming in bilinguals. Brain Res 1125: 116–131. 17113571 10.1016/j.brainres.2006.10.002

[100] Proverbio AM, Adorni R, Zani A (2009) Inferring native language from early bio-electrical activity. Biol Psychol 80: 52–63. 18378060 10.1016/j.biopsycho.2008.02.006

[101] Moreno EM, Rodriguez-Fornells A, Laine M (2008) Event-related potentials (ERPs) in the study of bilingual language processing. J Neurolinguistics 21: 477–508.

[102] Runnqvist E, Strijkers K, Sadat J, Costa A (2011) On the temporal and functional origin of l2 disadvantages in speech production: a critical review. Front Psychol 2: 1–8. 22203812 10.3389/fpsyg.2011.00379 PMC3241344

[103] Coderre EL, van Heuven WJB (2013) Modulations of the executive control network by stimulus onset asynchrony in a Stroop task. BMC Neurosci 14: 1–18. 23902451 10.1186/1471-2202-14-79 PMC3734141

[104] Novick JM, Kan IP, Trueswell JC, Thompson-Schill SL (2009) A case for conflict across multiple domains: Memory and language impairments following damage to ventrolateral prefrontal cortex. Cogn Neuropsychol 26: 527–567. 20183014 10.1080/02643290903519367 PMC3791076

[105] Novick JM, Trueswell JC, Thompson-Schill SL (2005) Cognitive control and parsing: Reexamining the role of Broca’s area in sentence comprehension. Cogn Affect Behav Neurosci 5: 263–281. 16396089 10.3758/cabn.5.3.263

[106] Thompson-Schill SL, Kurtz KJ, Gabrieli JDE (1998) Effects of Semantic and Associative Relatedness on Automatic Priming. J Mem Lang 38: 440–458.

[107] Braver TS, Paxton JL, Locke HS, Barch DM (2009) Flexible neural mechanisms of cognitive control within human prefrontal cortex. Proc Natl Acad Sci 106: 7351–7356. 19380750 10.1073/pnas.0808187106 PMC2678630

[108] Braver TS, Gray JR, Burgess GC (2007) Explaining the many varieties of working memory variation: Dual mechanisms of cognitive control. In: Conway ARA, Jarrold C, Kane MJ, Miyake A, Towse JN, editors. Variations in Working Memory. New York, NY: Oxford University Press. 76–106.

[109] De Pisapia N, Braver TS (2006) A model of dual control mechanisms through anterior cingulate and prefrontal cortex interactions. Neurocomputing 69: 1322–1326.

[110] Morales J, Gómez-Ariza CJ, Bajo MT (2013) Dual mechanisms of cognitive control in bilinguals and monolinguals. J Cogn Psychol 25: 531–546.

[111] Egner T, Hirsch J (2005) Cognitive control mechanisms resolve conflict through cortical amplification of task-relevant information. Nat Neurosci 8: 1784–1790. 16286928 10.1038/nn1594

[112] Vogel EK, Luck SJ (2000) The visual N1 component as an index of a discrimination process. Psychophysiology 37: 190–203. 10731769

